# Polyphenolic compounds as electron shuttles for sustainable energy utilization

**DOI:** 10.1186/s13068-019-1602-9

**Published:** 2019-11-18

**Authors:** Chung-Chuan Hsueh, Chia-Chyi Wu, Bor-Yann Chen

**Affiliations:** 1 Department of Chemical and Materials Engineering, National I-Lan University, I-Lan, 26047 Taiwan; 2 Department of Horticulture, National I-Lan University, I-Lan, 26047 Taiwan

**Keywords:** Electron shuttles, Polyphenolics, Microbial fuel cells, Antioxidants

## Abstract

For renewable and sustainable bioenergy utilization with cost-effectiveness, electron-shuttles (ESs) (or redox mediators (RMs)) act as electrochemical “catalysts” to enhance rates of redox reactions, catalytically accelerating electron transport efficiency for abiotic and biotic electrochemical reactions. ESs are popularly used in cellular respiratory systems, metabolisms in organisms, and widely applied to support global lives. Apparently, they are applicable to increase power-generating capabilities for energy utilization and/or fuel storage (i.e., dye-sensitized solar cell, batteries, and microbial fuel cells (MFCs)). This first-attempt review specifically deciphers the chemical structure association with characteristics of ESs, and discloses redox-mediating potentials of polyphenolics-abundant ESs via MFC modules. Moreover, to effectively convert electron-shuttling capabilities from non-sustainable antioxidant activities, environmental conditions to induce electrochemical mediation apparently play critical roles of great significance for bioenergy stimulation. For example, pH levels would significantly affect electrochemical potentials to be exhibited (e.g., alkaline pHs are electrochemically favorable for expression of such electron-shuttling characteristics). Regarding chemical structure effect, chemicals with *ortho*- and *para*-dihydroxyl substituents-bearing aromatics own convertible characteristics of non-renewable antioxidants and electrochemically catalytic ESs; however, ES capabilities of *meta*-dihydroxyl substituents can be evidently repressed due to lack of resonance effect in the structure for intermediate radical(s) during redox reaction. Moreover, this review provides conclusive remarks to elucidate the promising feasibility to identify whether such characteristics are non-renewable antioxidants or reversible ESs from natural polyphenols via cyclic voltammetry and MFC evaluation. Evidently, considering sustainable development, such electrochemically convertible polyphenolic species in plant extracts can be reversibly expressed for bioenergy-stimulating capabilities in MFCs under electrochemically favorable conditions.

## Background

**Fig. 1:**
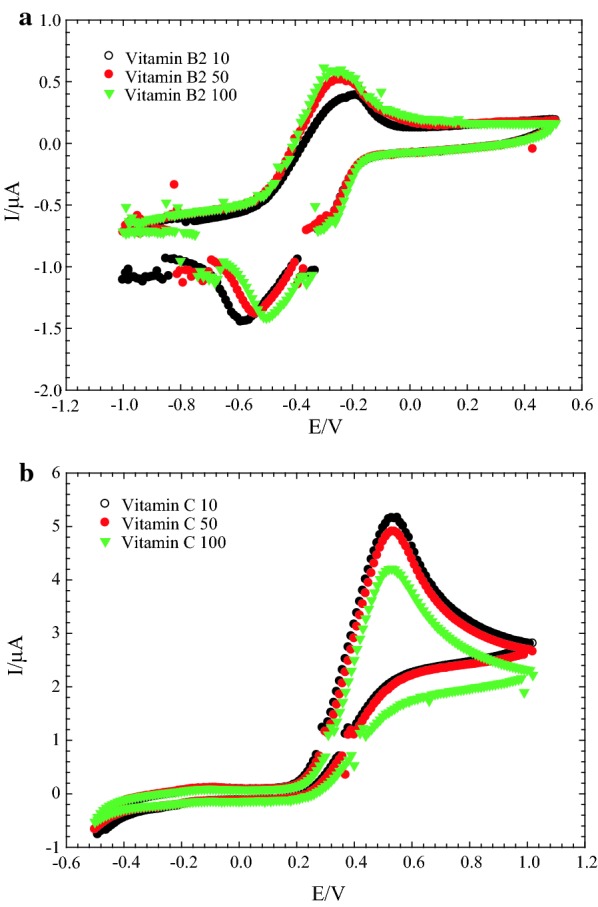
**a** Cyclic voltammetry of Vitamin B_2_ for 100 scan cycles, clearly indicating the persistence of two stable redox potential peaks of electron shuttle [10]. **b** Cyclic voltammogram of vitamin C (1000 mg/L) in PBS solution at scan rate 10 mV/s, indicating only one redox potential peak of antioxidant [10]

In this review, we aim to clarify some of the mysteries of the electron shuttles (ESs) used in sustainable bioenergy applications (e.g., electro-fermentation and electrochemical biorefining). In particular, increasing energy, and resource scarcity and ecological destruction have encouraged for bioremediation techniques and the use of renewable material and energy sources. Thus, the creation of innovative, practical “green” sustainable bioenergy applications is inevitably required. One example of this technology is the exploitation of the bioelectrochemical potential of electroactive microorganisms. Bioelectrochemical devices, such as microbial fuel cells (MFCs), can use synergistic/cooperative consortia of microbes as biocatalysts, and the electric current produced by the oxidation of organic compounds can be used for simultaneous bioelectricity generation and wastewater treatment [1–5]. In fact, MFCs are a promising platform to explore the electrochemical potential of bioresources for bioenergy and biorefinery uses. In an MFC, an anodic biofilm of bacteria is used to oxidize organic matter, which acts as nutrient source (i.e., the primary substrate), thus degrading pollutants or detoxifying inorganic contaminants co-metabolically. The protons generated in this process migrate through a proton exchange membrane toward the cathode in a double-chamber MFC, and the electrons overcome the interfacial mass transfer resistance on the anodic biofilm and pass through an external circuit to the cathode (e.g., air cathode), simultaneously generating bioelectricity. Therefore, bioelectricity generation occurs at the (bio)cathode along with water formation (i.e., ½O_2_ + 2H^+^ + 2e^−^ → H_2_O). There are several mechanisms for biofilm formation, direct electron transfer, mediator electron transport, and direct interspecies electron transfer that affect the operating efficiencies of MFCs [6]. To decrease the internal electron transfer resistance between electron acceptors and donors significantly, exogenous bioelectrochemical catalysts are commonly used; this allows the power-generating performance of MFCs to be increased. Moreover, electron transfer between microbes and from microbes to electron-accepting compounds can be facilitated using ESs. This is a promising way to decline the activation energy and increase efficient electron between the intracellular compartment and extracellular medium during microbial energy metabolism [7, 8]. That is, ESs are “electrochemical catalysts” that increase the reaction rate under appropriate environmental conditions. Because electrochemical catalysts (e.g., ESs) are not consumed during the catalytic reaction, they function reversibly, stably, and persistently. In particular, oxygen is a strong electron acceptor; anaerobic conditions are necessary to guarantee the effective redox-mediating behavior of ESs during electrochemical reactions. In the presence of ESs and the absence of strong electron acceptors (e.g., O_2_), electrochemically driven reactions and reductive biodegradation occur faster because such electrochemical catalyst provides an alternative reaction pathway with lower activation energy than that of the non-catalyzed mechanism. That is, ESs can speed up redox reactions by providing “a set of elementary steps” with more (bio)electrochemically favorable kinetics than those that exist in their absence. Thus, supplementing an ES to a MFC or bioreactor system that is not in steady state or equilibrium will speed up both the forward and reverse rates, allowing an equilibrium mixture to be achieved much faster than anticipated. Thus, ESs play a crucial role in increasing the electron transfer efficiency between electron donors and acceptors for sustainable bioenergy extraction. The electrochemical characteristics of ESs can be determined from cyclic voltammetry (CV) profiles, and the difference (Δ*E*) between the cathodic (reduction) potential (*E*_pc_) and anodic (oxidation) potential (*E*_pa_) is given by the Butler–Volmer equation and Cottrell equations: $i = \frac{{nFAC_{0} \sqrt D }}{{\sqrt {\pi t} }}$ and $E_{\text{pa}} - E_{\text{pc}} = \frac{{56.5\;{\text{mV}}}}{n}$, for an “*n*” electron process [9]. The observed redox potential peaks of vitamin B_2_ (riboflavin), a well-known ES, can be maintained over 100 CV cycles (Fig. 1a) [10]. In contrast, 100 CV cycles of the well-characterized antioxidant vitamin C only yielded an oxidation peak, as shown in Fig. 1b [10]. Furthermore, if the ratio of the peak currents of a test chemical approaches unity (i.e., *i*_pc_/*i*_pa_ ≈ 1 or the reduction and oxidation potentials are symmetric, where *i*_pc_ and *i*_pa_ are the cathodic and anodic peak currents for a reversible reaction, respectively), the chemical could be an ES with semi-reversible redox characteristics and electrochemical use (e.g., biorefinery and bioenergy uses) [11–13].

ESs can be either inorganic or organic compounds, such as iodide/triiodide [14] and Fe^3+^ [15], quinones [7], and vitamin B_2_ [16, 17]. Considering the need for environmental friendliness for sustainable development, organic compounds, both natural and synthetic, with low biotoxicities (e.g., polyphenols or polyhydroxyphenols) are favorable and promising for use as ESs compared to other inorganic species. In the following sections, we clarify several aspects of ESs not previously reported and suggest an optimal strategy for maximizing bioenergy extraction for sustainable development.

## Chemical characteristics of organic ESs

The chemical structure of an ES strongly affected whether electrons and radicals are stable and delocalized for bioenergy extraction. The chemical structures significantly influence chemical or physical characteristics (e.g., polar or non-polar) and functions (e.g., Lewis acids/electrophiles/oxidants or Lewis bases/nucleophiles/reductants) and reactivities. That is, the chemical structure directly determines the reactions and reactivity of an organic compound (e.g., strong or weak reducing or oxidizing activities). In particular, ESs can be used to stimulate the biodegradation of various pollutants and bioelectricity generation in MFCs [18, 19]. Therefore, it is important to disclose that chemical structure of organic ESs can be used as essential prerequisite to determine whether they can own the capabilities of ESs or not.

### Aromaticity

**Fig. 2:**
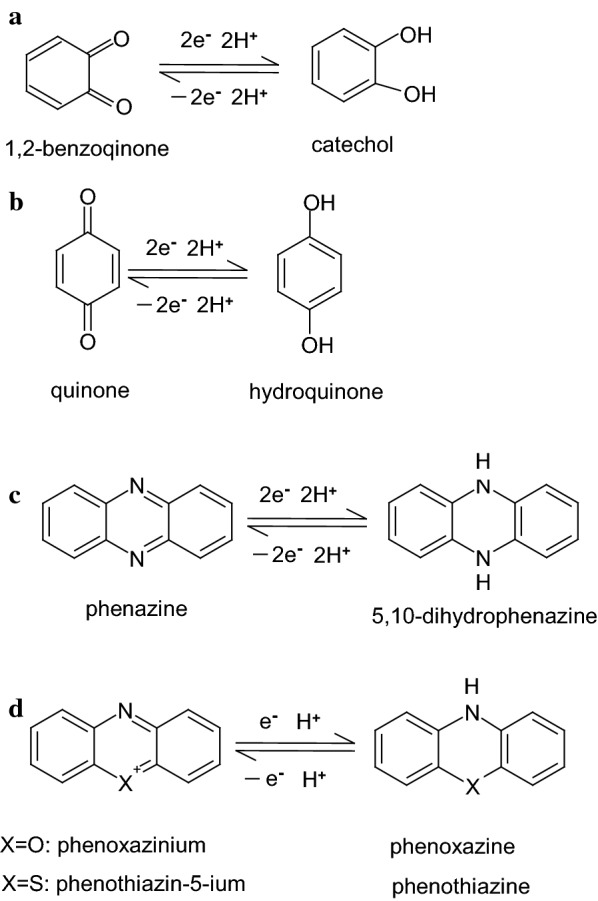
The redox reaction equations of electron shuttles

**Fig. 3:**
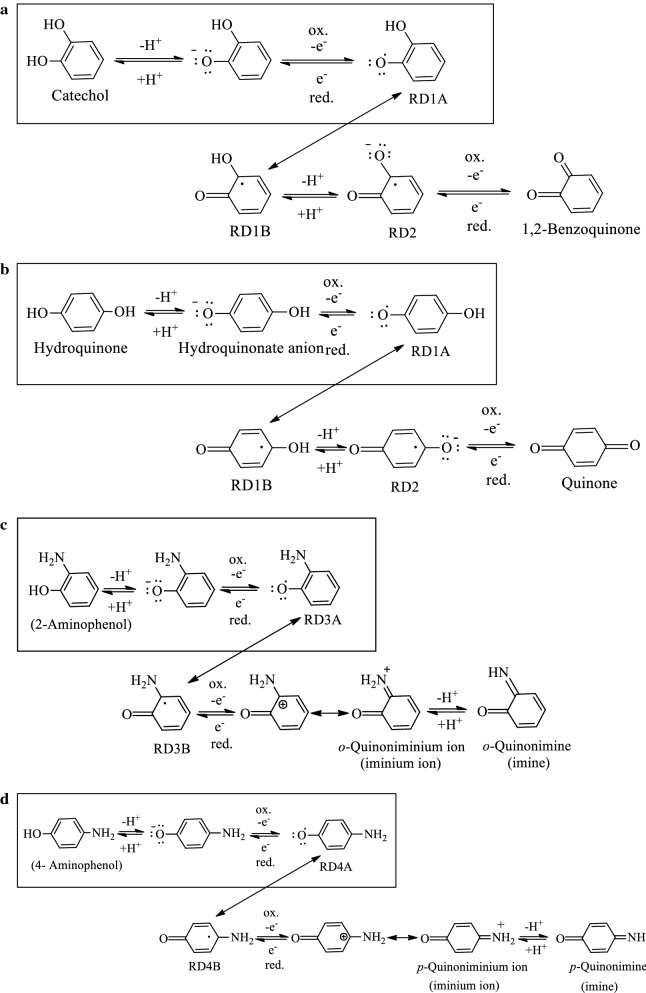
**a**–**d** proposed pathways of inter-conversion of hydroquinone/quinone and catechol/1,2-benzoquinone, 2-aminophenol/*o*-quinonimine, and 4-aminophenol/*p*-quinonimine, respectively (cited from [26]) (RD denoted resonance forms of intermediates)

Table 1 lists several different ESs (e.g., phenazine, riboflavin, phenoxazine, thionine, viologen, quinone, naphthoquinone, anthraquinone, and aminophenol); these compounds have been used as ESs in MFCs to enhance the biodecolorization of azo dyes, as well as bioelectricity generation [7, 16, 17, 20–27]. Because pollutant degradation (e.g., reductive biodegradation) and bioelectricity generation are both associated with electron transfer, the formation or addition of an appropriate ES species can enhance the reductive and oxidative capabilities of an MFC. Therefore, the supplementation of ESs can be a valuable strategy to enhance the efficiency for diverse redox-mediated treatments (e.g., electro-fermentation and reductive biodegradation). From the perspective of molecular structures, ESs must be able to form the structures that can delocalize electrons and radicals during the redox reactions, for example, by resonance effect owing to aromaticity. That is, ESs must be able to form stable intermediates/complexes rapidly to allow further electrochemical reactions to be initiated. Therefore, the activation energies of the redox reactions can be decreased, and the reaction rates will be increased significantly. As shown in Fig. 2 for the redox reactions of ESs, the radicals (i.e., unpaired electrons) formed on the aromatic rings of ESs during redox reactions are delocalized by significant resonance effects (e.g., RD1A ↔ RD1B as shown in Fig. 3a, b [26]), resulting in redox reversibility. However, the ability to delocalize electrons results in greater electrochemical stability, enhancing the reversible redox-mediating capabilities of these compounds [28, 29]. This delocalization of electrons, radicals, and charge in intermediates or “transition complexes” of the ESs with aromatic rings ensures their stable reversible electrochemical reactions. Due to the formation of radicals or the effective delocalization of charge in intermediate species, ESs are kinetically and electrochemically favorable electron mediators between oxidants and reductants [29]. Thus, aromaticity seems to be a critical characteristic of most ESs. Furthermore, the redox characteristics of aromatic ESs are affected by the electron withdrawing or donating nature of substituents, as well as their orientations, which strongly affect the reactivity and catalytic power [7].

**Table 1:** Some compounds used as electron shuttles (ESs) in microbial fuel cells (MFCs) or biological anaerobic decolorization (BD)

Redox mediators^a^ (ESs)	Applied condition^b^	References
Phenazine	MFC	[7]
	BD	[16]
Neutral red	BD	[16]
Phenazine ethosulphate	MFC	[20]
Safranine-O	MFC	[20]
Riboflavin	BD	[16]
	MFC	[17]
Phenoxazine		
Brilliant cresyl blue(s)	MFC	[20]
Gallocyanine	MFC	[20]
Resorufin	MFC	[20]
Azure A	MFC and BD	[21]
Azure C	MFC and BD	[21]
Methylene blue	MFC	[20]
New methylene blue	MFC	[20]
*N*,*N*-dimethyl-disulphonated thionine	MFC	[20]
Phenothiazinone	MFC	[20]
Thionine	MFC	[20]
	MFC and BD	[21, 22]
Toluidine blue-O	MFC	[20]
Benzyl viologen	BD	[16]
	MFC	[20]
Methyl viologen	BD	[16]
Viologen	MFC	[7]
Malachite green	MFC	[22]
2,6-Dichlorophenolindophenol	MFC	[20]
Cobalamine	MFC	[7]
	BD	[16]
Quinones series		
1,2-Benzoquinone	MFC and BD	[23]
Dopamine	MFC	[17]
Epinephrine	MFC	[17]
1,4-Benzoquinone	BD	[16]
	MFC	[24]
Naphthoquinone	MFC	[7]
	BD	[16]
	BD	[24]
	BD	[25]
Alizarin brilliant blue	MFC	[20]
Anthraquinone	MFC	[7]
	BD	[16]
	BD	[24]
	BD	[25]
2-Aminophenol	MFC and BD	[26]
1-Amino-2-naphthol	MFC and BD	[27]
4-Amino-1-naphthol	MFC and BD	[27]
Humic acid	MFC	[7]

^a^Redox mediator (RM) is also electron-shuttle (ES)

^b^BD and MFC denoted bio-decolorization and microbial fuel cell, respectively

### Substituent effects

As benzenes/polybenzenes contain a wide range of derivatives, easily obtained, and relatively inexpensive, they can be candidate sources of the practical ES species. Chen et al. [26] screened some model benzene derivatives with hydroxyl/amino and other substituents as possible ESs via CV analysis. The redox potential peaks of these benzene-based chemicals with different auxochromes (i.e., color-generating functional groups) were intentionally screened for possible electrochemical activity (e.g., antioxidant or ES). Such chemical with functional groups hydroxyl (–OH), amino (–NH_2_), sulfhydryl (–SH), and methoxy (–OCH_3_) carboxyl (–COOH) and sulfo (–SO_3_H)) groups was tested via CV analysis from − 0.8 to 0.6 V (Table 2). Thus, compounds such as 2-aminophenol (2-AP) and 4-aminophenol (4-AP) bearing these substituents are characterized as ESs (i.e., displayed *i*_pa_/*i*_pc_ ≈ 1). The CV results revealed that suitably placed hydroxyl and amino substituents (e.g., *ortho*- and *para*-) resulted in typical reduction and oxidation peaks characteristic of ESs [26]. As a matter of fact, because atoms O and N are more electronegative (3.44 and 3.04, respectively) than carbon, organic compounds containing these atoms could favorably act as oxidants (i.e., EAs) during the formation of oxidized forms (i.e., intermediates with low electron densities). However, when these atoms are bounded on the aromatic rings, the lone pair electrons on O/N can act as electron donors via resonance effects in the reduced forms (i.e., higher electron densities) [29]. That is, some polyphenolics or aromatic heterocyclic rings containing N or O are electrochemically active ESs (Table 1). In particular, if strongly activating electron donating substituents (e.g., –OH and –NH_2_) are present on an aromatic ring, radicals or charged ES intermediates located on O/N atom or on the carbon atoms attached O/N atom via resonance are electrochemically stabilized (Fig. 3a–d [26]), resulting in a decrease in the activation energy and a significant increase in the reaction rate.

**Table 2:** Different auxochromes on benzenes whether owning the capabilities to be electron shuttles

Auxochrome	Electron-mediating capabilities^a^
Hydroxyl group, –OH	+
Amino group, –NH_2_	+
Sulfhydryl group, –SH	−
Methoxy group, –OCH_3_	−
Carboxyl group, –COOH	−
Sulfo group, –SO_3_H	−

^a^**+**: with redox peaks on CV profile; **−**: without redox peaks on CV profile (cited from [26])

In fact, it was found that ESs with hydroxyl substituents are more stable than amino groups with respect to their electrochemical redox behaviors. For instance, compared to 1,2-diaminobenzene (also known as benzene-1,2-diamine), benzene-1,2-diol (catechol) shows superior redox reversibility during repeated CV cycling [23]. Moreover, if hydroxyl-bearing ES compounds are obtained from natural polyphenols (or polyhydroxyphenols), the ESs could be less toxic and more biocompatible than compounds containing amino groups, making them green and sustainable compounds for practical uses. For example, 4-AP is toxic to electroactive bacteria, and the bioelectricity-generating capability of an MFC inoculated with *Proteus hauseri* ZMd44 was significantly repressed by this compound [8, 30]; thus, the biotoxicities of the compounds must be assessed before use for biotechnological applications. That is, as hydroxyl groups are more electrochemically stable and reversible, and less toxic than amino groups, polyhydroxy derivatives of benzenes/polybenzenes (e.g., benzoquinone, naphthoquinones, anthraquinones, and other polyphenolic compounds) are good candidate ESs for sustainable applications [23]. Therefore, if polyphenolic compounds are obtained from natural sources to be ESs, they can be environmentally friendly and bioelectrochemically appropriate for diverse purposes (e.g., bioenergy and biofuel uses).

### Effect of substituent position

**Fig. 4:**
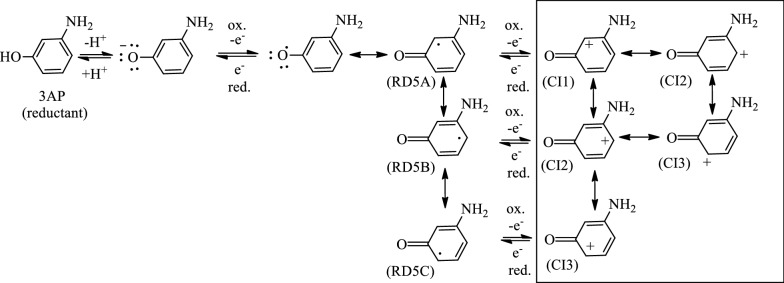
The resonance forms of the redox intermediates of 3-aminophenol (cited from [26])

The substitutional patterns (e.g., *meta*, *ortho*, or *para*) of functional groups on an aromatic ring affect both the reactivity and regioselectivity of electrophilic/nucleophilic aromatic substitutions via inductive and resonance effects [29]. Similar concepts may also be applicable to affect ES capabilities. For example, significant redox peaks were only observed in the CV profiles when the hydroxyl or amino groups were arranged at *ortho*- and *para*-positions on benzene-based redox mediators such as 2-AP, 4-AP, 1,2-diaminobenzene [23, 26], dopamine [17, 31], catechol, hydroquinone, caffeic acid, and paracetamol [31]. These compounds can show strong ES characteristics. Chen et al. [23, 26] employed 1,2-benzoquinone, 2-AP, and 1,2-diaminobenzene as ESs to improve the biodecolorization efficiency and increase the bioelectricity-generating capabilities of an MFC. In fact, Van der Zee et al. [16] and Bradley et al. [24] also used 1,4-benzoquinone to stimulate color removal and bioelectricity-generating capabilities in MFCs. Moreover, Guo et al. [17] recently reported that epinephrine and dopamine, which own *ortho*-dihydroxyl substituents, significantly enhance the bioelectricity production in MFCs. Possibly, the *ortho*- and *para*-positioned hydroxyl/amino groups in these compounds stabilize the radicals and charges in the intermediates (or transition states) of the ESs via resonance effects, thus mediating or accelerating redox reactions. That is, the radicals or charges could be delocalized on carbon atoms attached to hydroxyl or amino groups via resonance effects aided by the presence of the lone pairs on oxygen and nitrogen [26, 28, 29] (Fig. 3a–d modified from [26]). However, some compounds with hydroxyl or amino groups at the *meta*-position on phenyl rings can not exhibit redox potential peaks in their CV profiles, for example, 3-AP [26], resorcinol, and resveratrol [31]. Of course, these compounds are not ESs and only act as antioxidants because the hydroxyl or amino groups at the *meta*-position cannot stabilize radicals or charges in the redox intermediates (Fig. 4, cited from Ref. [26]). That is, the radicals or charges of the redox intermediates cannot be stabilized by delocalization on the O/N atom or the carbon attached to the O/N atom [29]. Although *meta*-dihydroxy aromatics (e.g., morin, resveratrol, and 3,5-dihydroxybenzoic acid, refer to Table 1 in [32]) are not ES compounds, they can still act as antioxidants for use as reductants in redox reactions. In summary, aromatic compounds with two or more hydroxyl or amino groups *ortho* or *para* to each other can provide a conjugated system for electron delocalization by resonance effect, making these species suitable mediators to shuttle electrons for further electron transport between electron donors and acceptors for electrochemical catalysis. As mentioned, hydroxyl-bearing compounds (e.g., polyphenols) are common antioxidants used in the treatment of pathogenic bacteria and environmental free radicals to increase cell survival and health. Natural polyphenolic compounds from edible and medicinal plants are appropriate sustainable ESs or reductants for use as redox enhancers in bioenergy and biofuel applications. Therefore, if ES compounds are obtained from natural sources, they can be environmentally friendly and bioelectrochemically appropriate for a range of purposes (e.g., bioenergy and biofuel uses).

## Natural polyphenolics as ESs

### Antioxidants as reductants or ESs

**Fig. 5:**
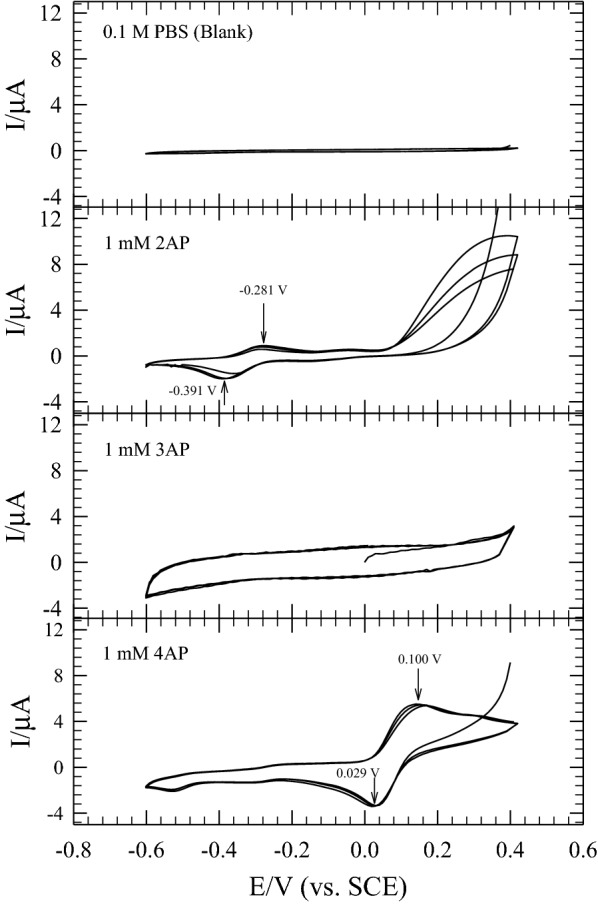
Three-scan cyclic voltammograms for redox processes of 1 mM aminophenol isomers (2-AP, 3-AP, 4-AP) in 0.1 M phosphate-buffered salines (PBS) at pH 7.0 (scan rate = 10 mV/s) (cited from [30])

Phenolic compounds are widely distributed in plants as secondary metabolites and, as antioxidants, can be used to scavenge free radicals and prevent oxidative stress. For many phenolic antioxidants [32], their reactions can be formulated as, R ⇄ O + *n*H^+^ +*n*e^−^, where R is the reductant (antioxidant) and O is the intermediate/product of oxidation. These phenolic antioxidants are necessary to sustain the normal metabolic functioning of plants. In addition, although many natural substances (e.g., edible flora and medicinal herbs) contain polyphenolic compounds, their ES and antioxidant capabilities can often be either synergistically or antagonistically affected and, sometimes, completely repressed by each other due to interactive complexity of the mixtures of these compounds. Consequently, it is difficult to distinguish the ES activity from antioxidant capability. For example, oxidation usually results in the generation of free radicals, which triggers further chain reactions that may damage individual cells or the whole organism. Antioxidants react with oxidants or radicals and, thus, inhibit or repress the oxidation of other more important species, such as biochemicals, nutrients, and cells. Antioxidants scavenge free radicals to prevent harmful reactions to be taken place. Thus, in this review, we discuss pure ES species to enable a comparison of ES and antioxidant behavior and exclude possible confounding effects of other chemical species. Moreover, whether an electroactive substance or chemical can act as either an ES or antioxidant strongly depends on the reversible electrochemical characteristics over multiple CV scans under specific conditions (e.g., the unstable and decaying profiles of 2-AP versus the stable reversible profiles of 4-AP in Fig. 5; [30]), even though both aminophenols may be ESs on the basis of their structure. As shown by the CV profiles (Fig. 5), the repeated oxidation and reduction of amino-bearing compounds (e.g., 2-AP) results in the gradual loss of electrochemical stability because of the repeated electrochemical oxidation and reduction, whereas hydroxyl substituents are more electrochemically stable [23, 26]. The reversible electrochemical behavior of ESs, as shown by their stable and reversible CV profiles, is characteristic of these “catalysts” crucial for their redox-mediating activity in pollutant biodegradation and power generation in MFCs. For example, catechol, an *ortho*-dihydroxy compound, is a typical biological redox mediator, and the compounds with catechol moieties are also found widely in the natural world. The redox stability of hydroxyl-containing compounds may explain why similar compounds (e.g., dopamine, epinephrine, norepinephrine, caffeic acid, and chlorogenic acid) are present in diverse organisms for bioenergy utilization. Catechol can be produced by the reversible two-electron, two-proton reduction of 1,2-benzoquinone (*E*° = + 795 mV versus (SHE), *E*_m_ (pH 7) = + 380 mV vs. SHE) (also see the CV profile of catechol in [23]). Thus, catechol is an ES with a suitable standard electrode potential (*E*°) for redox reactions, explaining its frequent presence in many organisms (Table 3, Fig. 3a). In addition, 100 repeated CV cycles on catechol at neutral pH show its reversible redox behavior and stable ES characteristics [23]. In contrast, antioxidants such as vitamin C (Fig. 1b) and vitamin E scavenge free radicals and form unstable intermediates, leading to gradual “decomposition” by electrooxidation, making antioxidants non-renewable reductants. In contrast, riboflavin (also known as vitamin B_2_) is a typical ES chemical species, remaining stable over multiple redox cycles (Fig. 1a). The oxidation of antioxidants under atmospheric conditions is unlikely to be reversed (i.e., to the original chemical species). That is, the loss of electrochemical characteristics cannot be prevented, resulting in gradually decaying redox peaks over repeated CV scans, as seen for 2-AP (Fig. 5).

**Table 3:** Typical standard reduction potentials of electron transfer carriers in organisms

Half-reaction	*E*^o^ (mV)
NAD^+^ + H^+^ + 2e^−^ → NADH	− 320
FAD + 2H^+^ + 2e^−^ → FADH_2_ (free coenzyme)	− 220
FAD + 2H^+^ + 2e^−^ → FADH_2_ (in flavoproteins)	~ 0
Coenzyme Q10	+ 100
Cytochrome C (Fe^3+^) + e^−^ → Cytochrome C (Fe^2+^)	+ 220

**Fig. 6:**
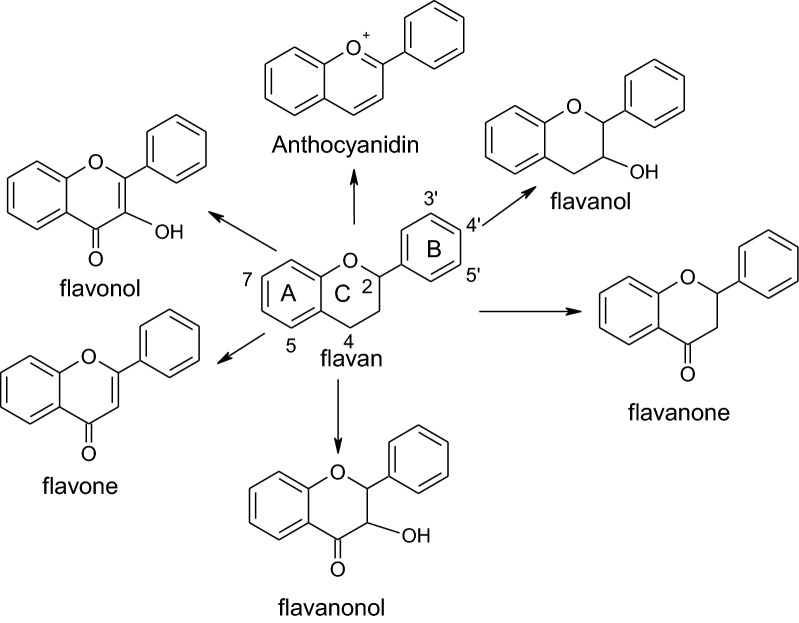
Classification of flavonoid subclasses

Polyphenolics are ubiquitous natural compounds, and these polyphenol/polyhydroxyphenol species have diverse chemical structures, and frequently show promise as antioxidants or reductants with cell-protecting effects [33–35]. In addition, although polyphenolics contain several hydroxyl groups, not all of the hydroxyl groups can be fully utilized for antioxidant or ES activity. For example, as reported by Lemannska et al. [36] and Firuzi et al. [37], hydroxyflavone isomers (see Fig. 6) have different antioxidant capabilities. For instance, the 3-OH (on the C ring) and 5-OH (on the A ring) isomers electrochemically favor high antioxidant activity, possibly because these hydroxyl groups are near the carbonyl group (C=O on C-4) allowing the formation of intramolecular hydrogen bonds (e.g.,  and  ) [38] to result in easier oxidation. In contrast, the 7-OH (on the A ring) and 4′-OH (on the B ring) isomers, where the hydroxyl groups are far from the carbonyl group, do not show antioxidant activities. Thus, despite having the same functional groups, these compounds have different electrochemical activities arising from orientation effects. These effects of the chemical structure on reactivity and catalysis can be analyzed by structure–activity relationships (SARs) [28, 39]. However, 1,2,3-trihydroxyl or 1,3,5-trihydroxyl arrangements are electrochemically favorable for high antioxidant activity; i.e., these compounds are good reductants [40]. That is, trihydroxyl-bearing flavonoids act as strong antioxidants, but simply function as reductants in MFCs for simultaneous pollutant degradation and bioelectricity generation [18, 41]. For example, the main polyphenolics in *Camellia* green tea, epicatechin gallate (ECG), epigallocatechin gallate (EGCG), and gallic acid (GA), have CV profiles that show significant oxidative potential peaks [41]; yet, the lack of significant reductive potential peaks (see Kilmartin and Hsu [42]) indicates that these compounds are not electrochemically viable as electron shuttles. However, they can still be as reductants to enhance the power density in MFCs [41]. Compared to the antioxidants GA and vitamin C, dopamine and epinephrine as ESs result in significant increases in power generation in MFCs [17]. Moreover, baicalins having 5,6-dihydroxy groups on the A ring display more significant reductive and oxidative potential peaks (i.e., *E*_pc_ and *E*_pa_) in their CV profiles than those bearing 5,6,7-trihydroxyl groups on the A ring [43]; however, baicaleins have higher free-radical-scavenging capabilities than baicalins [44]. In addition, when the CV profiles of test chemicals do not show reversible redox potential peaks, these chemicals can not maintain their chemical properties after redox cycling. This explains why CV analyses on medicinal herbal extracts usually showed gradually decaying redox potentials [41, 45]. That is, as CV profiles show an irreversible response curves, the compounds under study are easily converted to different chemical species after such repeated redox reactions. Furthermore, as reported by Danilewicz [46], catechol (H_2_Q) can undergo redox reactions under acidic conditions (pH 3.6) and an ideal CV profile showing distinct reduction and oxidation potential peaks is obtained. In addition, polyphenolics with *ortho*-dihydroxyl groups (e.g., epicatechin, catechin, 5-*O*-caffeoylquinic acid, caffeic acid, chlorogenic acid, rutin, and quercetin) also exhibited significant redox potential peaks on CV scanning [42, 47]. Of course, these polyphenolic compounds could not only be antioxidants but also ESs if appropriate environmental conditions are electrochemically satisfied. For example, at slightly alkaline pH, *Camellia* green tea and medicinal herbal extracts, which contain high antioxidant polyphenolic contents, could be converted as sustainable ES “catalysts” for renewable energy extraction [41, 45]. In contrast, polyphenolics having *meta*-dihydroxyl substituents on the phenyl rings (e.g., resorcinol and resveratrol) can only function electrochemically as antioxidants due to a lack of reductive potential peaks in their CV profiles [31]. Moreover, compared to baicalins, which contain 5,6-dihydroxy groups on the A ring, the redox potential peaks of wogonins and chrysin with 5,7-dihydroxy (i.e., *meta*-dihydroxy) groups on the A ring were almost absent because of the lack of the resonance structure required for ES behavior [43, 48, 49]. Evidently, the *meta*-dihydroxyl substituents are not electrochemically suitable for use as ES in cyclic bioenergy applications.

### Natural polyphenolics as ES species

**Fig. 7:**
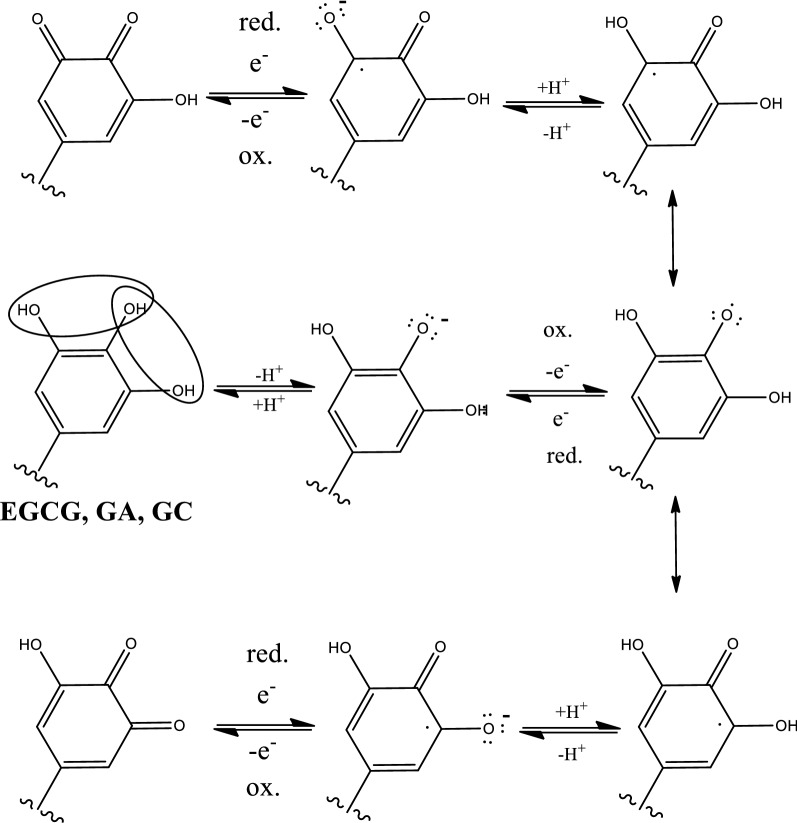
Proposed redox-mediating mechanisms of electron shuttle-major compositions of catechins (e.g., EGCG, GA, GC) in green tea extracts (cited from Fig. 5 of [41])

On the basis of the CV profiles of the polyphenolics discussed above, polyphenolic compounds with dihydroxyl groups *ortho*- or *para*- to each other could act as ESs. For example, humic acids (or humic substances) are well-known ESs in bioelectrochemical systems [7, 50–52]. As mentioned, phenolic compounds play crucial roles in plant development (e.g., lignin and pigment biosynthesis), and these compounds can be categorized as (A) polyhydroxyl derivatives of phenylpropanoids, (B) derivatives of quinones/naphthoquinones/anthraquinones or catechols, (C) polyhydroxyl derivatives of flavonoids, (D) polyhydroxyl derivatives of anthocyanidins, and (E) tannins (Table 4) [53–126]. Due to the earth’s species biodiversity, the levels of ESs vary significantly from plant to plant. However, some plant species contain abundant in ES chemicals (e.g., chlorogenic acid, rosmarinic acid, and verbascoside (acteoside)). For example, *Cynara scolymus* [58] and *Lonicera japonica* [62] contain 4.713% and 6.14% chlorogenic acid, respectively, and *Origanum majorana* L. and *Salvia officinalis* L. contain 15.15% and 17.85% rosmarinic acid, respectively [56]. In addition, *Orobanche rapum*-*genistae* [71] and *Plantago lanceolata* [85] contain 6% and 3–8% verbascoside (acteoside), respectively. *Salvia officinalis* L. and *Thymus vulgaris* L. contain different redox-mediating compounds. For example, they contain 1.98% and 1.40% caffeic acid, 3.77% and 7.57% carnosic acid, 8.61% and 7.65% luteolin-7-*O*-rutinose, and 2.52% and 1.72% quercetin 7-*O*-glucoside, respectively [56]. In particular, *Salvia officinalis* L. also possesses 1.22% chlorogenic acid and high levels of rosmarinic acid [56]. Moreover, *Salvia miltiorrhiza* contains 24.8% danshensu in its aqueous extracts [65]. Furthermore, 1.9% embelin could be extracted from *Embelia ribes* using hexane [94], and 12.4% shikonin derivatives could be extracted from a culture of *Lithospermum erythrorhizon* [100]. There are other cases of herbal plants containing hydrophobic (lipophilic) ESs that can be extracted by *n*-hexane. For example, high contents of naphthoquinone derivatives (e.g., 3′,3-biplumbagin, droserone, and plumbagin) could be obtained by extracting *Diospyros anisandra* [98] with *n*-hexane. In addition, 1.9% free and 4.5% total anthraquinones could be extracted from *Cassia occidentalis* roots [102], and *Rubia cordifolia* L. calluses produced two major anthraquinone: munjistin and purpurin, having a total anthraquinone yield of ca. 90% [113]. *Anemarrhena asphodeloides* Bunge contains plentiful isomangiferin, mangiferin, and neomangiferin [112]. Regarding flavonoid derivatives in plants, luteolin, quercetin, and their glycosides are also widely distributed in many plants. In fact, some plants contain very high quantities of flavonoids (e.g., quercetin and luteolin glycosides up to 21% and 38% for radicchio di Chioggia and radicchio di Treviso (both *Cichorium intybus*), respectively [60], and 9.38% luteolin-7-*O*-rutinose in *Origanum majorana* L. [56]). In addition, *Mentha* × *piperita* [73] contains 14.98% eriocitrin and 2.32% luteolin 7-*O*-rutinoside on a dry weight basis. Moreover, catechin and epicatechin were also widely distributed in many plants, such as tea plants, in high levels [118, 120]. In addition, wine-producing waste of *Vitis vinifera* [119] is also feasible to obtain ESs for MFCs. Similarly, because of the abundance of catechin and epicatechin, *Camellia* green tea could also have high antioxidant contents [127]. In addition, anthocyanidins (e.g., cyanidin and peonidin and their glycosides) are often present in red grapes, bilberries, blackberries, blueberries, cherries, cranberries, elderberries, hawberries, loganberries, açai berries, and raspberries. A relatively high percentage of total anthocyanins are found in bilberries (37.1%) compared to that of blueberries (17.6%) [125]. Further, ellagic acid is often found in nuts, berries, pears, peaches, plums, grapes, apples, and kiwi [126]. Thus, these polyphenolics-rich plants contain many electroactive chemical species that could serve as natural sources of electrochemically active species for biofuel applications. For example, the addition of ca. 2.5 g dry weight L^−1^ *Camellia* green tea extract as an ES considerably enhances power generation in MFCs [41, 45]. In fact, the bioelectricity-generating performance of supplemented MFCs has been increased by 143–167% compared to a supplement-free MFC (from 11.40–12.56 to 30.51 mW/m^2^ for *Camellia* green tea extract supplementation [127]). This increase in bioelectricity generation is promising for bioelectrochemical biofuel utilization. For example, the serial brewing of *Camellia* tea extracts at different degrees of fermentation (e.g., green, oolong, and black tea) by a comparative assessment of the DPPH free radical-scavenging activities showed the outstanding benefit of using *Camellia* tea waste for cost-effective bioenergy applications [127]. As Chen et al. revealed [41], for many different types of *Camellia* tea extracts, the antioxidant activity, total polyphenolics contents (e.g., EGCG, GA, and GC), and ES capabilities are strongly interrelated. As a matter of fact, *Camellia* tea has been shown to have an effect on human health, especially concerning to age-related deterioration of the brain [128]. Thus, *Camellia* tea extracts are promising for enhancing bioenergy generation over several cycles, and 47% and 45% activity remained after 5 and 4 cycles when using unfermented *Camellia* green tea and partially fermented oolong tea, respectively [127]. In contrast, because black tea (e.g., Pu’er tea) is a completely fermented tea, its DPPH free radical-scavenging activity is significantly lower, < 10% after three cycles of tea brewing. Thus, *Camellia* green tea extract is the most bioelectrochemically favorable ES extract for bioenergy applications. As mentioned, extracts of these polyphenolics-rich plants can act as either antioxidants or ESs under appropriate conditions. For instance, catechins (i.e., the main components of *Camellia* tea extracts) can be either antioxidants (i.e., oxidation peak dominant) or ESs (i.e., nearly symmetric oxidation and reduction potentials), as shown in the CV profiles of some catechins [42] and various *Camellia* tea extracts [45]. Because of the condition dependence of the ES and antioxidant behavior of catechins (e.g., EGCG, GA, and GC), both the electron-mediating mechanism of an ES (e.g., Fig. 7 obtained from [41]) and that of an antioxidant [129] are feasible. For example, catechin has been shown to act as an ES at pH > 8 and an antioxidant at pH < 6 [41]. In addition, Xu et al. [130] reported that the power density of an MFC was considerably enhanced by the addition of extracts of the anthocyanin abundant *Lycium ruthenicum Murr*. As shown by the ES mechanism (Figs. 2, 3), ES or antioxidant behavior is dependent on the ability to form stable phenolic radicals. Thus, the environmental conditions are crucial in determining whether an ES or antioxidant undergoes stable redox reactions. Moreover, a recent literature [131] revealed that the interactions of bacterial consortia in the hot springs of the Yellowstone National Park resulted in the synergistic evolution of a microbial community under hostile conditions (e.g., sulfur-containing hot springs), resulting in the ability to utilize formaldehyde as an ES. Consequently, control of the biotic or abiotic environment can convert an ES to an antioxidant and vice versa. Although some criteria of obtaining an electrochemically favorable environment for ES activity have been reported [7], the understanding of the change between ES and antioxidant behavior still remains limited. It has been proposed that the environmental conditions for the conversion of an ES to an antioxidant are strongly dependent on the redox reversibility of the derived intermediate radical species. However, the typical characteristics of antioxidants can be easily expressed because antioxidants can be gradually oxidized, leading to progressive attenuation in CV profiles, as discussed earlier. Thus, some polyphenolic compounds could function as reversible ESs under specific controlled abiotic or biotic conditions. Considering the practicality of using plant polyphenolics, natural polyphenolics are usually less biotoxic to receptor microbes and show higher electrochemical activities as ESs to stimulate the bioelectricity generation of electrochemically active microorganisms. For example, 3 to 5 times serially brewed extracts of *Camellia* green tea still exhibited significant antioxidant activity [127] because of its abundant antioxidant chemical species with low biotoxicity. This indicates that, with toxicity attenuation, even waste medicinal herbs have sufficient potential reuse in practical and sustainable bioenergy applications. Although the environmental conditions required for reversible ES characteristics are not always satisfied [7, 15], these natural polyphenolic compounds can function as antioxidants (i.e., reductants) for non-sustainable use (e.g., the enhancement of the rates and efficiencies of redox reactions in reductive biodegradation and bioelectricity generation). However, for the environmentally friendly and sustainable use of polyphenolic compounds, the electrochemical activities of ESs are still favorable if such polyphenolics have the capability to switch roles between antioxidant and ES (e.g., *ortho* or *para* dihydroxyl-bearing aromatic compounds).

**Table 4:** Polyhydroxyphenols (polyphenols) as electron shuttles in plants

Model polyphenols	Contents in plant species	Refs.
A. Derivatives of phenylpropanoids		
Caffeic acid	1.05–62.84 mg in 100 g dry samples of *Echinacea purpurea*	[53]
	773 mg phenolics (as caffeic acid) in 5 g sample of *Ilex paraguariensis*	[54]
	0.62 mg/g of *Monarda fistulosa* L.	[55]
	7.99–19.75 mg in 100 g dry samples of *Salvia apiana*	[53]
	1.98% in *Salvia officinalis* L.	[56]
	1.4% in *Thymus vulgaris* L.	[56]
Caffeoylquinic acid	~ 11 mg/g dry sample of *Akebia quinata*	[57]
	0.740% in dry sample of *Cynara scolymus*	[58]
	773 mg phenolics (as caffeoylquinic acid)/5 g sample of *Ilex paraguariensis*	[54]
	723 mg/100 g fresh samples of *Vallaris glabra*	[59]
Monocaffeoyl tartaric acid	223 μg caffeic acid (as monocaffeoyl tartaric acid)/g in fresh leaves of *Cichorium intybus* L.	[60]
Calceolarioside	~1 mg/g dry sample of *Akebia quinata*	[57]
Chlorogenic acid	566.5 μg of caffeic acid (as chlorogenic acid)/g fresh leaves of *Cichorium intybus*	[60]
	4.713% in dry sample of *Cynara scolymus*	[58]
	2.60% in dry samples of *Eucommia ulmoides*	[61]
	6.14% in dried plant samples of *Lonicera japonica*	[62]
	1.22% in *Salvia officinalis* L.	[56]
	0.22% in dry leaves weight of *Taraxacum officinale* Wigg	[63]
	1.02% in dry leaves weight of *Vaccinum arctostaphylos* L.	[63]
Cichoric acid (chicoric acid)	1535 μg/g fresh leaves of *Cichorium intybus*	[60]
	0.41% in dry aerial parts weight of *Chicorium intybus*	[63]
	42.37–258.70 mg caffeic acid (as chicoric acid)/100 g dry samples of *Echinacea purpurea*	[53]
	88.5 mg in 100 g fresh weight of *Ocimum basilicum* L.	[64]
	0.77% in dry leaves weight of *Taraxacum officinale* Wigg	[63]
Cynarin	1.689% in dry sample of *Cynara scolymus*	[58]
Danshensu	24.8% in crude aqueous extract of *Salvia miltiorrhiza*	[65]
Echinacoside	42.71% in 210 g crude phenyl ethanol glycosides extract from dried and sliced rhizomes of *Cistanche tubulosa* (6.0 kg)	[66]
Forsythiaside	16.7 mg from 500 g *Forsythia suspense*	[67]
Gravolenic acid	*Ruta graveolen*	[68, 69]
Nordihydroguaiaretic acid (NDGA)	0.0898% in the dried weight of *Larrea divaricata*	[70]
Orobanchoside	3% from *Orobanche rapum*-*genistae*	[71]
Pedicularioside	70 mg from 13.5 kg *Buddleia lindleyan*	[72]
Rosmarinic acid	9.30 mg/g *S. africana*-*lutea*	[55]
	3.68% from dry weight basis in 40 *Mentha* × *piperita* Clones	[73]
	622.28 mg caffeic acid (as rosmarinic acid)/100 g dry samples of *Melissa officinalis*	[53]
	15.15% in *Origanum majorana* L.	[56]
	8% of rosemary extract from 50 g dried leaves of *Rosmarinus officinalis* L.	[74]
	4.8 g from dried roots of *Salvia miltiorrhiza* (32 kg)	[75]
	17.85% *in Salvia officinalis* L	[56]
Salvianolic acid A	0.7 g from the dried roots of *Salvia miltiorrhiza* (32 kg)	[75]
	11.96 g residue from the dried roots of *Salvia miltiorrhiza* (30 kg)	[76]
Salvianolic acid B (lithospermic acid B)	2.06 g from the dried roots of *Salvia miltiorrhiza* (32 kg)	[75]
	33.93 mg/g in *Salvia miltiorrhiza (Danshen)*	[77]
Salvianolic acid C	0.4 g from dried roots of S *Salvia miltiorrhiza (Danshen)* (32 kg)	[75]
Tubuloside (A, B, C, D)	Tubuloside A (II) 200 mg; Tubuloside B (VI) 100 mg; Tubuloside C (VII) 60 mg; Tubuloside D (VIII) 65 mg from fresh whole plants of *Cistanche tubulosa* (Schrenk) (22 kg)	[78]
Verbascoside (acteoside)	0.009% (pure compound) from dried leaves (1 kg) of *Buddleja globosa*	[79]
	16 g isolated from dried and ground leaves of *Buddleja cordata* (2.3 kg)	[80]
	361.2 mg from the fresh whole plants (246 g) of *Byblis liniflora Salisb.*	[81]
	14.27% in 210 g crude phenyl ethanol glycosides extract from dried and sliced rhizomes of *Cistanche tubulosa* (6.0 kg)	[66]
	0.50 g from 35 g *Lantana camara*	[82]
	0.43 g from 35 g dry *Lippia. javanica*	[82]
	1.7% from dry weight of *Olea europaea*	[83]
	6% from the dried plant (*Orobanche rapum*-*genistae)*	[71]
	11.3 mg/mL (*n*-butanol extracts from dried and powdered leaves of *Phlomis* species (100 g))	[84]
	3–8% in *Plantago lanceolata*	[85]
	0.612 mg/g dry extract of *Verbascum mallophorum*	[86]
	0.18–3.03% of the dry weight *Verbascum* leaves	[87]
	30.31 mg/g dry weight of *Verbascum nigrum*	[87]
	~ 3.7% in MeOH extract from ground aerial parts (1.3 kg) of *Verbena officinalis*	[88]
	13.28 mg/g dry weight of *Verbascum phoeniceum* species	[87]
	15.79 mg/g dry weight of *Verbascum xanthophoeniceum*	[87]
B-1. Quinones and derivatives		
Hydroxyechinofuran B	Hydroxyechinofuran B (15 mg) from 1 g of fresh hairy roots of *Lithospermum erythrorbizon*	[89]
6-(4,7-Hydroxy-heptyl) cyclohex-2,5-diene-ene-1,4-dione (6-(4,7-hydroxy-heptyl) quinone)	the new quinone isolated from ten grams of crude ethyl acetate extract of 500 g *Pergularia daemia* leaves	[90]
Scabequinone	~ 7 × 10^−3^ % from fresh *Cyperus distans* weight	[91]
2,6-Dimethoxyquinone	~ 0.002% based on root of *Rauwolfia vomitoria*	[92]
	1.1% of aqueous methanol fraction from 15 g dried stems of *Tibouchina pulchra*	[93]
2,5-Dihydroxy-3-undecyl-para-benzoquinone (Embelin)	1.9 g embelin from 100 g of *Embelia ribes* extracted using hexane	[94]
B-2. Catechol and derivatives		
Catechol (1,2-dihydroxybenzene).	0.1–0.2 g of crude catechol from 100 g of dry scales of pigmented onion scales (*Allium cepa*)	[95]
Protocatechuic acid (3,4-dihydroxybenzoic acid)	~ 0.1 g/100 g dry scales of pigmented onion scales (*Allium cepa*)	[96]
Carnosic acid	3.77*% in Salvia officinalis* L.	[56]
	7.57% in *Thymus vulgaris* L.	[56]
Dopaol beta-d-glucoside (2-(3,4-dihydroxyphenyl) ethyl β-d-gluco-pyranoside)	~ 3 mg/g dry sample of *Akebia quinata*	[57]
Horminone	0.4 g from about 1 kg of the dried whole plant (aerial parts and roots of *Salvia lanata*)	[97]
B-3. Naphthoquinones and derivatives		
3′3-Biplumbagin	~ 10% from the n-hexane extracts of stem bark (20 g) of *Diospyros anisandra*	[98]
Diosquinone	120 μg from 5 kg of the dried root of *Diospyros mespiliformis*	[99]
Droserone	~ 70% from the n-hexane extracts of stem bark (20 g) of *Diospyros anisandra*	[98]
Maritinone	~ 5% from the n-hexane extracts of stem bark (20 g) *Diospyros anisandra*	[98]
Plumbagin	~ 30% from the n-hexanic extracts obtained from Powdered stem bark (20 g) *Diospyros anisandra*	[98]
	34 mg from 5 kg of the dried root of *Diospyros mespiliformis*	[99]
Shikonin (derivatives)	12.4% shikonin derivatives in cells corresponding to 3 g dry wt. inoculated on 1 L of the medium of *Lithospermum erythrorhizon*	[100]
Vitamin K	Spinach, swiss chard, lettuce and *Brassica* vegetables (such as cabbage, kale, cauliflower, broccoli, and brussels sprouts), avocados, kiwifruit and grapes	[101]
B-4. Anthraquinones and derivatives	1.9% free and 4.5% total anthaquinones in *Cassia occidentalis* roots	[102]
	hydroxyanthraquinones indicated in *Cassia angustifolia* Vahl, *Cassia senna* Linn. and *Cassia tora*	[103]
	0.04 to 3.8, 5.9 and 36 mg total anthraquinones per kg fresh weight in peas, cabbage, lettuce and beans, respectively	[103]
Alizarin (1,2-dihydroxyanthraquinone)	39 mg from *Rubia cordifolia* L. root powder (3 kg)	[104]
	147.9 and 216.8 *μ*g/g dry weight of *Rubia peregrina* in roots and callus, respectively	[105]
	939.6 and 335.2 *μ*g/g dry weight of *Rubia tinctorum* in roots and callus, respectively	[105]
Chrysophanol	140 mg from powdered roots of *Rumex nepalensis* (2 kg)	[106]
1,3,8-Trihydroxy-6-methylanthracene-9,10-dione (Emodin, Emodol, Schuttgelb)	0.1 g/8 kg (0.0012%) of *Aster tataricus*	[107]
	79 mg from *Cassia obtusifolia* seeds (4.9 kg)	[108]
	2.03 mg/g in *Frangula alnus* bark	[109]
	16.2 mg from 928 g of the ethanol extract (9% (g/g)) of the dried stem and roots of *Polygonum hypoleucum* Ohwi	[110]
	105 mg from powdered roots of *Rumex nepalensis* (2 kg)	[106]
	0.31% (w/w) from shade-dried plant samples of *Ventilago madraspatana* (500 g)	[111]
Emodin-8-*O*-β-d-glucopyranoside	17.4 mg from 928 g of the ethanol extract (9% (g/g)) of *Polygonum hypoleucum* Ohwi)	[110]
	35 mg from powdered roots of *Rumex nepalensis* (2 kg)	[106]
Endocrocin	7 mg from powdered roots of *Rumex nepalensis* (2 kg)	[106]
Isomangiferin	15 mg/g in *Anemarrhena asphodeloides* Bunge	[112]
	2 mg/g in *Anemarrhenae Rhizoma*)	[112]
Mangiferin	31 mg/g in *Anemarrhena asphodeloides* Bunge	[112]
	16 mg/g in *Anemarrhenae Rhizoma*	[112]
Neomangiferin	16 mg/g in *Anemarrhena asphodeloides* Bunge	[112]
	8 mg/g in *Anemarrhenae Rhizoma*	[112]
Munjistin	Two major anthraquinones (munjistin and purpurin) representing 90% of the total anthraquinone yield from *Rubia cordifolia* L.calluses	[113]
Physcion	0.11 mg/g in *Frangula rupestris* bark (Scop.)	[109]
	8 mg from powdered roots of *Rumex nepalensis* (2 kg)	[106]
	0.22% (w/w) from shade-dried plant samples of *Ventilago madraspatana* (500 g)	[111]
Purpurin	Two major anthraquinones (munjistin and purpurin) representing 90% of the total anthraquinone yield from *Rubia cordifolia* L.calluses	[113]
C. Derivatives of flavonoids		
Azaleatin	2 × 10^−3^ % from *Plumbago indica*	[114]
Catechin	10.9 (mg/g) catechin from *Abies pindrow*	[115]
	75 (mg/g) in catechu resin chunks (*Acacia catechu*)	[116]
	2.67 (mg/g) from *Cedrus deodara*	[115]
	358 mg from 100 g dry matter in Chardonnay seeds	[117]
	127 mg from 100 g dry matter in Merlot seeds,	[117]
	12 mg from 100 g dry matter in Muscadine seeds	[117]
	the total catechins content in green tea ranges from 9 to 117 mg/g of dry weight of green tea leaves	[118]
	6.15(mg/g) from *Pinus gerardiana*	[115]
	13.3 (mg/g) from *Pinus roxburghii*	[115]
	5.78 (mg/g) from *Pinus wallichiana*	[115]
	12.7 mg isolated from 928 g of the ethanol extract (9% (g/g)) of *Polygonum hypoleucum* Ohwi	[110]
	21.8 (mg/g) from *Taxus fuana*	[115]
	3.6 mg/g (dry weight) of red wine wastes from *Vitis vinifera*	[119]
	56 mg/g (dry weight) of white wine wastes from *Vitis vinifera*	[119]
	total catechins, including EGCG, ECG, EC and EGC, accounted for 10.1 g/100 g dry longjing tea leaves	[120]
Cedeodarin	3.23 (mg/g) from *Cedrus deodara*	[115]
Epicatechin	15 (mg/g) in catechu resin chunks (*Acacia catechu*)	[116]
	421 mg in 100 g dry matter in Chardonnay seeds	[117]
	115 mg in 100 g dry matter in Merlot seeds	[117]
	96 mg in 100 g dry matter of Muscadine seeds	[117]
	3.1 mg/g dry weight of red wine wastes from *Vitis Vinifera*	[119]
	100 mg/g dry weight of white wine wastes from *Vitis Vinifera*	[119]
	7.5 mg isolated from 928 g of EP the ethanol extract (9% (g/g)) of *Polygonum hypoleucum* Ohwi)	[110]
	Epicatechin < 11 mg/g tea	[121]
Epicatechin-3-*O*-gallate	21 (mg/g) in catechu leaves (*Acacia catechu*)	[116]
Eriocitrin	14.98% (with a concentration range of 6.6–15.0%) in *Mentha* × *piperita*	[73]
Isomangiferin	15 mg/g in *Anemarrhena asphodeloides* Bunge	[112]
	2 mg/g in *Anemarrhenae rhizoma*	[112]
Mangiferin	31 mg/g in *Anemarrhena asphodeloides* Bunge)	[112]
	16 mg/g in *Anemarrhenae Rhizoma*	[112]
Luteolin-7-*O*-glucuronide	1650 μg rutin (as luteolin-7-*O*-glucuronide)/g fresh leaves of *Cichorium intybus*	[60]
	Quercetin and luteolin glycosides up to 21% and 38% for radicchio di Chioggia and radicchio di Treviso respectively	[60]
Luteolin-7-*O*-rutinose	0.420% in dry sample of *Cynara scolymus* L.	[58]
	9.38% in *Origanum majorana* L.	[56]
	8.61% *in Salvia officinalis* L.	[56]
	7.65% in Thymus vulgaris L.	[56]
	2.32% (dry Weight Basis) in 40 *Mentha* × *piperita* Clones	[73]
Quercetin	9.3 mg from the air-dried and powered rhizomes of *Alpinia officinarum* (3.2 kg)	[123]
	Quercetin (1.6 g/8 kg) (0.02%) in *Aster tataricus*	[107]
	Quercetin and luteolin glycosides up to 21% and 38% for radicchio di Chioggia and radicchio di Treviso, respectively	[60]
	Rutin ~ 190 mg/g) and quercetin ~ 14 mg/g) from *Sophora japonica* L. buds	[122]
Quercetin 3-*O*-glucuronide	1055 μg as rutin/g in fresh leaves of *Cichorium intybus*	[60]
Quercetin 7-*O*-glucoside	2.52% in *Salvia officinalis* L.	[56]
	1.72% in *Thymus vulgaris* L.	[56]
Dihydroquercetin (taxifolin)	5.98 (mg/g) in *Cedrus deodara*	[115]
	2.65 (mg/g) in *Pinus roxburghii*	[115]
Theaflavin	1.58 g/100 g dry leaves of qimen black tea	[124]
D. Anthocyanidin	Cyanidin and peonidin glycoside representing 37.1% of total anthocyanins in bilberries, and 17.6% in blueberries	[125]
Cyanidin	Cyanidin and peonidin glycoside representing 37.1% of total anthocyanins in bilberries, and 17.6% in blueberries	[125]
Cyanidin 3-*O*-glucoside	536 μg keracyanin (as cyanidin 3-*O*-glucoside)/g in fresh leaves of *Cichorium intybus*	[60]
	Red grapes, bilberry, blackberry, blueberry, cherry, cranberry, elderberry, hawthorn, loganberry, açai berry and raspberry	[125]
Cyanidin 3-*O*-(6″malonyl)-glucoside	4790 μg keracyanin (as cyanidin 3-*O*-(6″malonyl)-glucoside)/g in fresh leaves of *Cichorium intybus*	[60]
Petunidin (Petunidol; Myrtillidin)	red grapes, bilberry, blackberry, blueberry, cherry, chokeberries, cranberry, elderberry, hawthorn, loganberry, açai berry and raspberry	[125]
E. Tannin; ellagic acid	Strawberries (630 μg/g dry wt), raspberries (1500 μg/g dry wt), blackberries (1500 μg/g dry wt), walnuts (590 μg/g dry wt), pecans (330 μg/g dry wt), cranberries (120 μg/g dry wt)	[126]

### Feasible ES flavonoids

Many natural polyphenolics, in particular the flavonoids in edible flora, are ubiquitous, relatively inexpensive, highly biocompatible, and generated in large quantities. Thus, under appropriate conditions (e.g., alkaline pH), these compounds could be highly environmentally friendly ESs for sustainable applications. The skeleton of flavonoids contains three six-membered rings (i.e., A, B and C rings; Fig. 6). As mentioned above, dihydroxyl groups are located at different positions in different rings, the antioxidant and ES capabilities vary significantly, because of synergistic effects or noncooperative structural effects, *ortho*-dihydroxy compounds, such as flavonoids, show different reactivities when the functional groups are arranged on different rings (i.e., the A, B, or C rings in Fig. 6), as reported by Lemannska et al. [36]. Moreover, 6,7-dihydroxy and 7,8-dihydroxy substitution on the A ring results in much stronger antioxidant capabilities than 3′,4′-dihydroxy substitution on the B ring. In addition, as Makhotkina and Kilmartin reported the strong oxidation potential of a catechin having 5,7-dihydroxyl groups on the A ring at ca. 850 mV, whereas reversible redox peaks corresponding to the *ortho*-dihydroxy groups on the B ring were observed at about 380 and 400 mV [12]. Firuzi et al. [37] also revealed that redox peaks on CV profiles of taxifolin, rutin, catechin, baicalein, quercetin, fisetin, and myricetin with redox peaks corresponding to the *ortho*-dihydroxy groups on the B rings appearing at ca. 300–460 mV in the CV profile. These results suggest that 3′,4′-dihydroxy groups on the B ring result in reversible redox behavior. Therefore, flavonoids with *ortho*-dihydroxy groups on the B ring are potential electrochemically favorable ESs. Obviously, the chemical compounds bearing at least one *ortho*-dihydroxy group on the benzene ring could be ESs. Some common flavonoids with *ortho*-dihydroxy or *para*-dihydroxy groups that are potential ESs are listed in Table 5 [56, 132–136]. In addition, glycosides or oligomeric flavonoids could also be used as ESs under the appropriate conditions to trigger ES activity [7, 15]. In particular, by changing the operating variables to convert non-sustainable antioxidants to renewable ES catalysts for sustainable applications, the productivities of bioenergy processes (e.g., electro-fermentation, biorefining, and bioremediation) can be maximized, especially if the structural criteria for ESs are satisfied.

**Table 5:** Comparative list of typical feasible ES-behaved flavonoids

Classify	Feasible ES-behaved flavonoids
Anthocyanidins	Arrabidin^e^, 3′-hydroxyarrabidin^e^, carajurin^e^, cyanidin^b^, 5-methylcyanidin^e^, cyanidin-3-O-glucoside (chrysanthemin), cyanidin-3-O-rutinoside, cyanidin-3-O-malonylglucoside, cyaniding-3-O-galactoside-5-O-glucoside, cyanin^b^, europinidin^e^, kuromanin^b^, petunidin^e^, petunidin-3-glucoside, riccionidin A^e^
Flavans	Butiniflavan^d^, luteoliflavan^d^
Flavanol (flavan-3,4-diols)	Catechin^b^, epicatechin^b^, epicatechin gallate^b^, epicatechin-5- or -7-*O*-xylofuranoside^c^, epigallocatechin gallate^b^, fisetinidol^c^, epifisetinidol^c^, mesquitol^d^, gleditsin (fisetinidol-4β-ol)^c^, theaflavin, 4β-carboxymethylepicatechin (dryopteric acid)^d^
Flavanones	Carthamidin^a^, carthamidin-7-*O*-d-glucuronide^a^, dihydrobaicalin^a^, dihydronorwogonin^a^, dihydrorehderianin I^a^, eriodictyol^b^, isocarthamidin-7-*O*-d-glucuronide^a^, naringenin^b^,
Flavanonols	3,6,7,2′,6′-pentahydroxyflavanone^a^, taxifolin (dihydroquercetin)^b^
Flavone	Amentoflavone^a^, baicalin^a^, baicalein-7-*O*-d-glucopyranside^a^, baicalein-7-*O*-l-rhamnoside^a^, cirsilineol^a^, 5,6-dihydroxy-7-*O*-glucosideflavone^a^, 5,8-dihydroxy-6,7-dimethoxyflavone^a^, ganhuangenin^a^, 6-hydroxyflavone^a^, ikonnikoside I^a^, luteolin^b^, orientin^b^, norwogonin^a^, norwogonin-7-*O*-d-glucuronopyranoside^a^, scutellaprostin C^a^, scutellaprostin F^a^, scutellarin^a^, scutellarein-7-*O*-d-glucopyranside^a^, 5,6,2′,6′-tetrahydroxy-7,8-dimethoxyflavone^a^, (2*S*)5,7,3′,4′-tetrahydroxyflavone (eriodictyol)^a^, 5,6,2′-trihydroxy-7,8-dimethoxyflavone^a^, 5,8,2′-trihydroxy-7-methoxyflavone^a^, 5,8,2′-trihydroxy-7-*O*-d-glucopyranoside^a^, 5,8,2′-trihydroxy-6,7-dimethoxyflavone^a^, 5,6,2′-trihydroxy-7,8,6′-trimethoxyflavone^a^, 5,7,2′,3′-tetrahydroxyflavone^a^
Flavonol	Fisetin^b^, isorhamnetin^b^, quercetin^b^, quercetin 3-*O*-glucuronide (miquelianin), quercetin-7-*O*-glucoside, quercetin-3-*O*-malonylglucoside, rutin^b^, quercetrin^b^, rhamnetin^b^, Quercetin-7-o-glucoside^f^,
Proanthocyanidin or dimeric flavonoids^c^,	Probutinidin^d^, procyanidins B1-B4^d^, profisetinidins^c^,
Others or glycosides or esters, or oligomeric flavonoids^d^	Epimopanol^c^, mopanin^c^, mopanol^c^, peltogynin^c^, peltogynol^c^, trimeric profisetinidins^c^

^a^ [131], ^b^ [132], ^c^ [133], ^d^ [134], ^e^ [135], ^f^ [56]

### Methods to extract ESs from natural bioresources

**Fig. 8:**
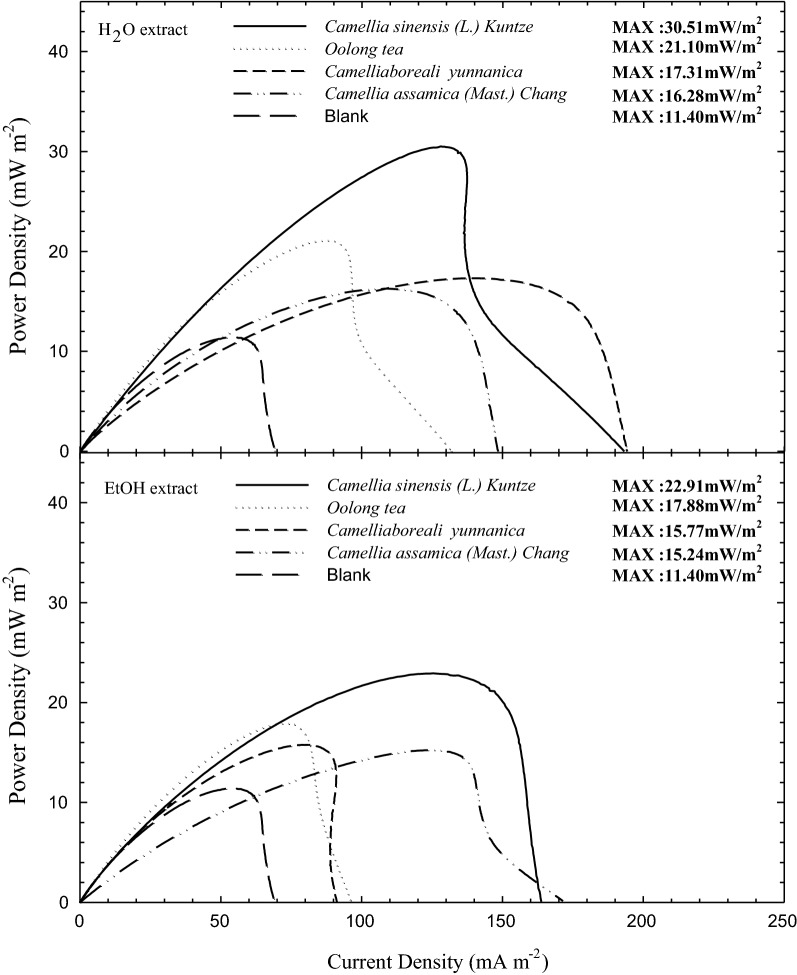
Comparison of bioelectricity generating capabilities in *Shewanella* sp. inoculated single-chamber MFCs with supplementation of water and ethanol extracts for various degree of fermented teas (cited from [46])

One crucial question is how to effectively extract electroactive natural polyphenolics from their source. The extraction method and parameters (e.g., temperature) affect the compounds obtained (e.g., relative ratios of antioxidants and ESs) [45]. For instance, among the various tea extracts, aqueous extracts are more electroactive than ethanol extracts for bioenergy applications (Fig. 8) [43], possibly because of the more favorable ES extraction. This is shown by a comparison of the enhancement in the power generated (units: mW/m^2^) when using different tea extracts in MFCs: *Camellia sinensis* (L.) Kuntze (green tea) (30.51) > Oolong tea (21.10) > *Camellia boreali*-*yunnanica* (17.31) > *Camellia assamica* (Mast.) Chang (16.28) > blank (11.40). In contrast, the use of ethanol extracts resulted in the following ranking: *Camellia sinensis* (L.) Kuntze (green tea) (22.91) > Oolong tea (17.88) > *Camellia boreali yunnanica* (Dianhong) (15.77) ≈ *Camellia assamica* (Mast.) Chang (Pu’er) (15.24) > blank (11.40) [43]. This comparison clearly shows that the use of different solvents extracts distinct electrochemically functioning species in different amounts from *Camellia* tea leaves. Thus, appropriate solvent selection for the extraction of electroactive ESs and electrochemical components is of course crucial. Furthermore, if the selected solvent is “generally recognized as safe” (e.g., water or ethanol), the harvested herbal extracts should be more biocompatible, inevitably increasing their potential range of applications. Regarding the condition-dependent electroactive characteristics (i.e., antioxidant or ES behavior), aqueous extracts of *Camellia* green tea seems to be more electrochemically promising for ES function than ethanol extracts [45], although some reports [137] have mentioned that 100% ethanol extraction resulted in the most abundant antioxidant polyphenolic content. That is, water extraction might be feasible to effectively harvest the most abundant ES polyphenolics in *Camellia* green tea. That is, an optimal temperature to extract maximal amounts and the most electrochemically functioning ES compounds could be expected for herbal extracts. However, the use of an excessively high temperature may decompose such electroactive compounds, leading to a significant attenuation in the electrochemical activities. For example, as Chen et al. [45] indicated, the optimal temperature to extract the maximum electrochemically active components from *Syzygium aromaticum* and *Camellia* green tea was ca. 65 °C, and temperatures higher than 65 °C hampered the extraction of electroactive compounds, possibly because of thermal decomposition.

## Environmental conditions to convert natural polyphenolic compounds to ESs

Regarding the convertible nature or natural polyphenolics, several factors influence the conversion of natural polyphenolics to ESs within living organisms: (1) range of reduction potentials, (2) pH, (3) water solubility, (4) biotoxicity to electrochemically active bacteria, and (5) possible biodegradability to utilize carbon as an energy source.

### Potential of polyphenolic compounds as ESs for MFCs

**Fig. 9:**
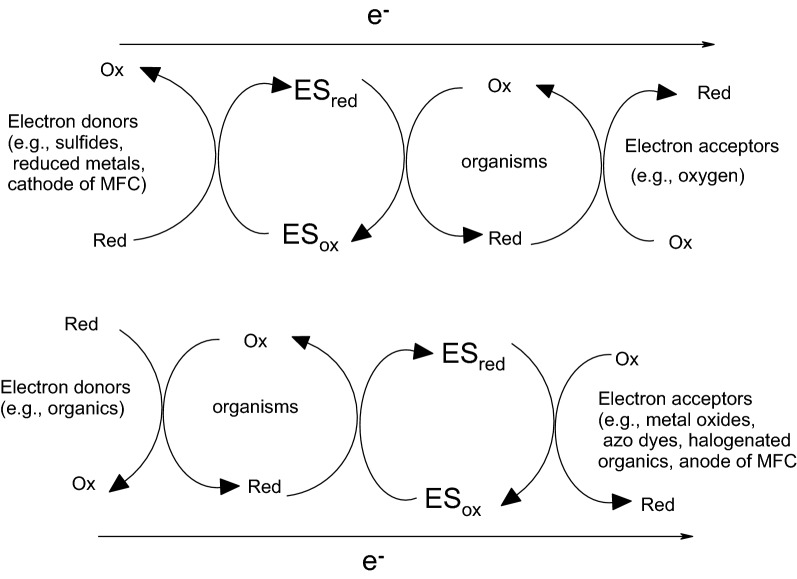
Schematics of electron transfer pathways with aid of redox reactions of electron shuttles in organisms (modified from [7])

Crucially, the redox potentials of these compounds are between those of the electron donor (ED) and electron acceptor (EA) (e.g., $E_{\text{ES}}^{0} = \frac{1}{2}\left( {E_{\text{ED}}^{0} + E_{\text{EA}}^{0} } \right)$), making these ESs electrochemically favorable for mediating electron transfer. To mediate electron transfer between electron donors and acceptors, the reduction potentials (*E*_ES_) of the candidate ES should be lower than the potential (*E*_EA_) of the oxidant (EA) and higher than the potential (*E*_ED_) of the reductants (ED). That is, if ∆*E* = *E*_EA_ – *E*_ES_ > 0, it is electrochemically favorable for the reduced form of the ES to reduce the oxidant (EA). Similarly, if ∆*E* = *E*_ES_ − *E*_ED_ > 0 [7, 16, 50], the reductant (ED) will reduce the oxidized ES (Fig. 9; [7]). If the oxidized electron shuttle (ES_ox_) is considered to be reduced by biological reduction systems, then the standard reduction potential (*E*_ES_) of the ES should not be lower than that of the biological reduction system (Table 3). That is, ES_ox_ can be reduced by the biological systems. Because of this specific reductive potential, an ES can electrochemically function only under appropriate environmental conditions. Polyphenolic compounds are usually weakly acidic compounds, so the reduction potential can be affected by the concentration and pH, as indicated by the Nernst equation. For example, quinone (Q) + 2H^+^ + 2e^−^ → hydroquinone (H_2_Q), and the reduction potential ($E = E^{0} - \frac{RT}{2F}\ln \left( {\frac{{[{\text{H}}_{2} {\text{Q}}]}}{{[{\text{Q}}][{\text{H}}^{ + } ]^{2} }}} \right)$) of quinones varies with the concentration of chemical species and pH [138]. Similarly, the oxidation potential, as shown by the Nernst equation, is also affected by the chemical and proton concentrations. As a matter of fact, the first *E*_pa1_ (measured by cyclic voltammetry and listed in Table 2 of [133]) of some polyphenolic compounds ranged from 0.30 to 0.72 V at pH 3.6–4.0 [133], whereas the first *E*_pa1_ of other polyphenolic compounds ranged from 0.087 to 0.236 V at concentrations of 0.01–0.05 mM and nearly neutral pH [42]. Thus, the redox potentials of these polyphenolics could be adjusted by varying the proton and chemical concentrations to achieve the correct electrochemical activity over the threshold for bioenergy stimulation before use in MFCs. With appropriate supplementation of chemical species, the electrochemical activities of polyphenolic compounds could be significantly promoted for efficient bioenergy extraction (e.g., increased the electrochemical content and conversion of antioxidants to ESs).

### Effect of pH on the redox potentials of polyphenolic compounds

**Fig. 10:**
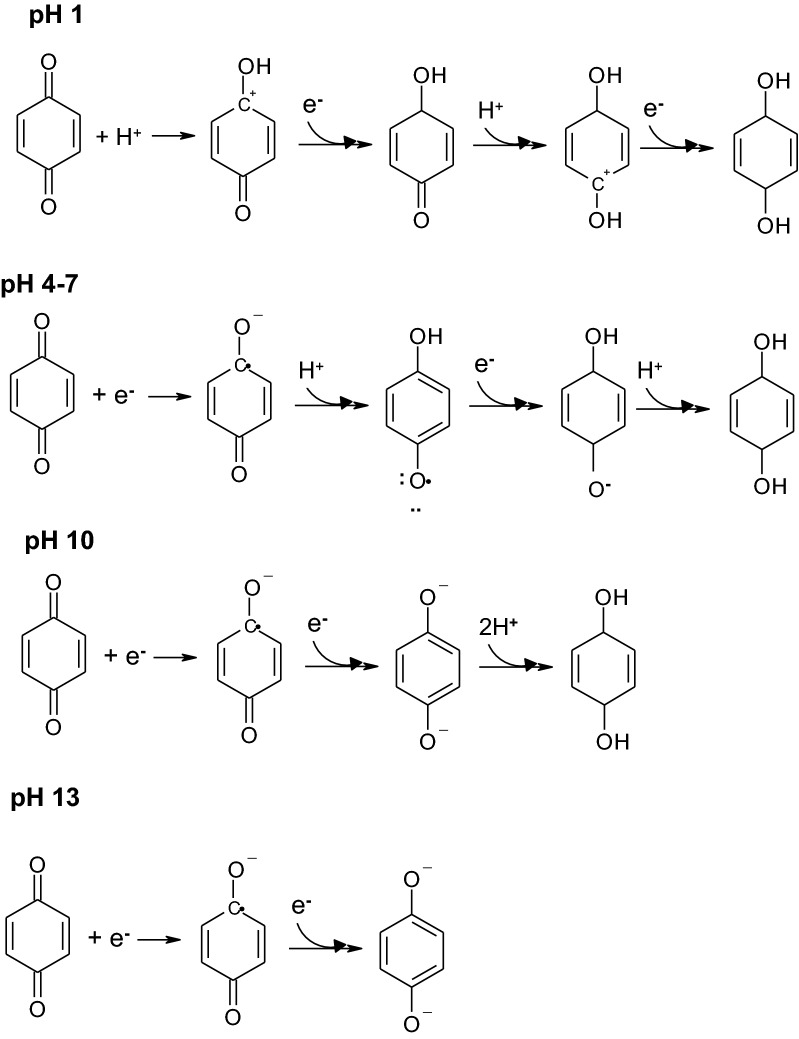
Comparative pathways of reductions and protonations of quinones at different pHs (modified from [139])

As discussed, the redox potentials of polyphenolic compounds are strongly influenced by the pH and concentrations of chemical species. For example, alternative redox reactions can also occur at different pH values, as shown in Fig. 10 (modified from [139]). For an example of a pH-dependent electrochemical reaction, at highly acidic pH (e.g., pH 1.0), quinones are first protonated and then reduced, as reported by Wedege et al. [139]. In contrast, at slightly acidic or neutral pHs (e.g., pH 4.0–7.0), quinones are reduced first and then protonated, and repeated reduction and protonation can occur sequentially. At alkaline pH (e.g., pH = 10), quinones are reduced twice in sequence of deprotonation, resulting in different electrochemical characteristics. Thus, pH is a feasible control variable affecting the conversion of antioxidant to ES “catalysts” in bioenergy applications. As revealed by the Nernst equation, the pH-dependent reduction potentials of some polyphenolic compounds (e.g., caffeic acid, catechin, and chlorogenic acid) are slightly decreased as the pH increases from 7.0 to 10.0 [140]. Moreover, as observed in their CV profiles, the *E*_pa_ and *E*_pc_ values of catechin, chlorogenic acid, epicatechin, epigallocatechin gallate, and gallic acid can shift toward lower values as the pH increases from 5.0 to 6.0 [141] according to Le Chatelier’s principle and the Nernst equation. It has also been reported that the reduction potentials of some polyphenolics (i.e., luteolin and catechol) linearly decrease with increasing pH [142, 143]. In addition, Chen et al. [45] studied extracts of *Camellia sinesis* (L.) Kuntze (green tea) and *Syzygium aromaticum* at a slightly alkaline pH (approximately 10) and found that this pH is more appropriate to augment bioenergy expression. That is, the appropriate manipulation of the pH and ES concentrations can result in polyphenolic compounds with electrochemically favorable redox potentials and low biotoxicities for renewable bioenergy extraction in bioelectrochemical reactors (e.g., MFCs).

### Solubilities of polyphenolic compounds as ESs in MFCs

Because MFCs are usually operated in an aqueous phases, ESs should be highly water soluble to attenuate the electron transfer resistance. Thus, slightly water-soluble anthraquinones (e.g., water solubility < 1 g/L at 23 °C) can be sulfonated to be anthraquinone-2-sulfonates (AQS) or anthraquinone-2-sulfonic acids to increase the water solubility. In fact, these modifications increase the water solubility; they can remarkably decrease the mass transfer resistance to enhance electron transport efficiency in wastewater bioremediation and bioelectricity generation in MFCs [25, 144, 145]. Furthermore, these modified ESs could be biotoxic and environmentally unfriendly. However, because natural polyphenolic and flavonoid ESs with low toxicity are often weakly acidic, they can be dissolved easily in alkaline aqueous solution or with initial addition of small volume of ethanol. Moreover, monosaccharides can be bound to polyphenolics or flavonoids to form glycosides, significantly increasing their water solubilities [146].

### Toxicity of polyphenolic compounds in MFCs

Although polyphenol and flavonoid-containing herbal extracts (e.g., *Camellia* green tea) usually have low biotoxicity, a few natural polyphenols and flavonoids may be still toxic. Thus, any antibacterial characteristics must be attenuated prior to bioenergy applications. In fact, the antibacterial activity of these compounds is often the reason to be considered for uses in medicine. For example, of the compounds extracted from *Scutellaria baicalensis*, the biotoxicities of baicaleins were higher than those of baicalins; thus, baicaleins are not suitable for direct applications to MFCs [43]. In addition, the toxicity of aqueous *Camellia* tea extracts to microorganisms is possibly lower than that of methanol extracts, even if the methanol is completely removed via freeze-drying (i.e., lyophilization), resulting in a high-quality tea extract (data not shown). In fact, the biotoxicity of *Camellia* or medicinal herbs (e.g., *Lonicera japonica*, *Citrus reticulate,* and *Scutellaria baicalensis*) extracts can be declined by electrochemical treatment (e.g., serial CV scans); thus, the power-generating capabilities of MFCs could be increased by the use of processed or CV-treated herbal extracts [45].

### Biodegradability and use as possible energy sources in MFCs

Regarding natural polyphenolic compounds, for example, the main components of *Camellia* green tea (EGCG, ECG, GA, and GC) could act as nutrient sources for microorganisms, as well as ESs and antioxidants, indicating that it is not only the ES activity that stimulates bioelectricity generation in MFCs [41, 45]. However, the conversion efficiency of nutrient source to be electrochemical catalyst for energy generation is not known, and further research is still required.

### Overall performance assessment

In summary, some suggestions of significance are provided for further exploration of natural ESs for renewable bioenergy utilization. As Chen et al. [45] reported, although *Camellia* green tea extract contains 10-times the total polyphenolic contents of other promising electroactive edible flora and medicinal herbs, only a threefold increase in power density is achieved when *Camellia* extracts are used in MFCs. That is, the maximal increase in bioenergy extraction is only ca. 30%. Detailed studies to enhance bioenergy mining and conversion are inevitably required from an engineering perspective. (a) The biotoxicity potency: medicinal herb extracts could have biotoxic/inhibitory effects upon bioreceptors (e.g., electroactive bacteria), thereby repressing the bioelectricity-generating performance of MFCs. (b) Electron transport limitation: the efficiency of bioelectricity generation is affected not only by the characteristics and concentrations of electroactive bacteria but also by the electrode characteristics and other factors affecting mass transfer (e.g., liquid–solid interface electron transport resistance) (c) Antioxidant/redox mediator conversion: The repeated CV responses revealed that the electroactive components of herbal extracts are gradually attenuated, indicating that these compounds are antioxidants rather than ES and revealing the relatively low ES contents of herbal plants. (d) CV and MFC conditions: Cyclic voltammetry is an abiotic redox process, but these redox reactions may not be directly related to the metabolic functions of bacteria. That is, the bioelectricity generating characteristics of MFCs may place a metabolic burden on electroactive bacteria, limiting MFC performance. (e) Inconsistency of redox activity: due to the different components in herbal extracts, there is a lack in consistency in the redox potentials required for effective, reversible electrochemical activity. The unbalanced electrochemical characteristics of the existing antioxidant species reflect irreversible redox characteristics, i.e., free radical scavenging as functioning reductants, and the reversible electron transfer activities of ES species may be not fully expressed or may be even masked. (f) Biodegradation: Some compounds may be consumed by the bacteria as nutrients rather than used as ESs, resulting in lower power generation performance than anticipated. Furthermore, if the electrochemical conditions are appropriate, ESs could behave as antioxidants. However, many antioxidants might not be capable of becoming ES species because of inadequate (electro)-chemical reactivity or orientation for such redox-mediating behavior. These all state that antioxidant and ES polyphenolics may be present; however, specific environmental conditions to maximize their bioenergy extracting efficiency still require further exploration.

## Conclusion

Under electrochemically suitable conditions, *ortho*- or *para*-dihydroxy-bearing polyphenolics are potential electroactive ESs for increasing the electron transfer rates of pollutant biodegradation and bioelectricity generation in MFCs. In particular, plant polyphenolics are abundant renewable bioresource for bioenergy applications. Because polyhydroxy flavonoids are ubiquitous, highly diverse, relatively nontoxic, and biocompatible, they are ecologically promising ESs for bioenergy technology. Because of the weakly acidic nature of polyphenolics, electrochemical manipulations can control the relative contents of chemical species or pH levels of polyphenolics, altering the redox potentials of polyphenolics for bioenergy generation or pollutant biodegradation. In addition, water solubility (i.e., a basic criterion to decrease electron transfer resistance in aqueous media for organisms and MFCs) can be increased at slightly alkaline pH conditions. These points provide a guide to create environmentally friendly and renewable bioenergy extraction processes.

## Data Availability

All data generated or analyzed during this study are included in this article.

## Abbreviations

2-AP: 2-aminophenol
4-AP: 4-aminophenol
AQS: anthraquinone-2-sulfonates
CV: cyclic voltammetry
EA: electron acceptor (i.e., oxidant)
ED: electron donor (i.e., reductant)
ES: electron shuttle
ESox: oxidized ES
$E_{\text{ES}}^{0}$: standard reduction potential of ES
*E*_ES_: reduction potential of ES
$E_{\text{EA}}^{0}$: standard reduction potential of EA
*E*_EA_: reduction potential of EA
$E_{\text{ED}}^{0}$: standard reduction potential of ED
*E*_ED_: reduction potential of ED
*E*_pa_: anodic (oxidative) potential
*E*_pc_: cathodic (reductive) potential
ECG: epicatechin gallate
EGCG: epigallocatechin gallate
GA: gallic acid
H_2_Q: catechol or hydroquinone
Q: quinone
MFC: microbial fuel cell
RM: redox mediator

## References

[1] Logan BE, Hamelers B, Rozendal R, Schröder U, Keller J, Freguia S (2006). Microbial fuel cells: methodology and technology. Environ Sci Technol.

[2] Ren H, Lee HS, Chae J (2012). Miniaturizing microbial fuel cells for potential portable power sources: promises and challenges. Microfluid Nanofluidics..

[3] Rabaey K, Verstraete W (2005). Microbial fuel cells: novel biotechnology for energy generation. Trends in Biotechnol..

[4] Rozendal RA, Hamelers HVM, Rabaey K, Keller J, Buisman CJN (2008). Towards practical implementation of bioelectrochemical wastewater treatment. Trends Biotechnol..

[5] Rozendal RA, Leone E, Keller J, Rabaey K (2009). Efficient hydrogen peroxide generation from organic matter in a bioelectrochemical system. Electrochem Commun.

[6] Kumar R, Singh L, Zularisam AW (2016). Exoelectrogens: recent advances in molecular drivers involved in extracellular electron transfer and strategies used to improve it for microbial fuel cell applications. Renew Sustain Energy Rev.

[7] Watanabe K, Manefield M, Lee M, Kouzuma A (2009). Electron shuttles in biotechnology. Curr Opin Biotechnol..

[8] Ng IS, Hsueh CC, Chen BY (2017). Electron transport phenomena of electroactive bacteria in microbial fuel cells: a review of *Proteus hauseri*. Bioresour Bioprocess..

[9] Bard AJ, Faulkner LR (2001). Electrochemical Methods: fundamentals and applications.

[10] Chen BY, Hsueh CC (2016). Deciphering electron shuttles for bioremediation and beyond. Am J Chem Eng..

[11] Mabbott GA (1983). An introduction to cyclic voltammetry. J Chem Educ.

[12] Makhotkina O, Kilmartin PA (2010). The use of cyclic voltammetry for wine analysis: determination of polyphenols and free sulfur dioxide. Anal Chim Acta.

[13] Elgrishi N, Rountree KJ, McCarthy BD, Rountree ES, Eisenhart TT, Dempsey JL (2018). A practical beginner’s guide to cyclic voltammetry. J Chem Educ.

[14] Boschloo G, Hagfeldt A (2009). Characteristics of the iodide/triiodide redox mediator in dye-sensitized solar cells. Acc Chem Res.

[15] Wang Q, Huang L, Pan Y, Quan X, Puma GL (2017). Impact of Fe(III) as an effective electron-shuttle mediator for enhanced Cr(VI) reduction in microbial fuel cells: reduction of diffusional resistances and cathode over potentials. J Hazard Mater.

[16] Van der Zee FP, Cervantes FJ (2009). Impact and application of electron shuttles on the redox (bio)transformation of contaminants: a review. Biotechnol Adv.

[17] Guo LL, Qin LJ, Xu B, Wang XZ, Hsueh CC, Chen BY (2019). Deciphering electron-shuttling characteristics of epinephrine and dopamine for bioenergy extraction using microbial fuel cells. Biochem Eng J.

[18] Luo H, Liu G, Zhang R, Jin S (2009). Phenol degradation in microbial fuel cells. Chem Eng J.

[19] Franks AE, Nevin KP (2010). Microbial Fuel Cells. A Current Review. Energies..

[20] Roller SD, Bennetto HP, Delaney GM, Mason JR, Stirling JL, Thurston CF (1984). Electron-transfer coupling in microbial fuel cells: 1. comparison of redox-mediator reduction rates and respiratory rates of bacteria. J Chem Technol Biotechnol..

[21] Chen BY, Xu B, Yueh PL, Han K, Qin LJ, Hsueh CC (2015). Deciphering electron-shuttling characteristics of thionine-based textile dyes in microbial fuel cells. J Taiwan Inst Chem Eng..

[22] Chen BY, Xu B, Qin LJ, Lan JCW, Hsueh CC (2014). Exploring redox-mediating characteristics of textile dye-bearing microbial fuel cells: thionin and malachite green. Bioresour Technol.

[23] Chen BY, Hsueh CC, Liu SQ, Ng IS, Wang YM (2013). Deciphering mediating characteristics of decolorized intermediates for reductive decolorization and bioelectricity generation. Bioresour Technol.

[24] Bradley RW, Bombelli P, Rowden SJL, Howel CJ (2012). Biological photovoltaics: intra- and extra-cellular electron transport by cyanobacteria. Biochem Soc Trans.

[25] Rau J, Knackmuss HJ, Stolz A (2002). Effects of different quinoid redox mediators on the anaerobic reduction of azo dyes by bacteria. Environ Sci Technol.

[26] Chen BY, Hsueh CC, Liu SQ, Hung JY, Qiao Y, Yueh PL (2013). Unveiling characteristics of dye-bearing microbial fuel cells for energy and materials recycling: redox mediators. Int J Hydrogen Energy..

[27] Xu B, Chen BY, Hsueh CC, Qin LJ, Chang CT (2014). Deciphering characteristics of bicyclic aromatics-mediators for reductive decolorization and bioelectricity generation. Bioresour Technol.

[28] Farhoosh R, Johnny S, Asnaashari M, Molaahmadibahraseman N, Sharif A (2016). Structure–antioxidant activity relationships of *o*-hydroxyl, *o*-methoxy, and alkyl ester derivatives of *p*-hydroxybenzoic acid. Food Chem.

[29] McMurry J. Chapter 9: aromatic compounds and Chapter 18: amine and heterocycles. In: McMurry J, editor. Organic chemistry with biological applications. 3rd ed. Cengage Learning. 2015.

[30] Chen BY, Wang YM, Ng IS, Liu SQ, Hung JY (2012). Deciphering simultaneous bioelectricity generation and dye decolorization using *Proteus hauseri*. J Biosci Bioeng.

[31] Sýs M, Metelka R, Frangu A, Vytřas K, Arbneshi T (2017). Electrochemical study of *Trametes versicolor* laccase compatibility to different polyphenolic substrates. Chemosensors..

[32] Kilmartin PA (2001). Forum method communication, electrochemical detection of natural antioxidants: principles and protocols. Antioxid Redox Signal.

[33] Perron NR, Brumaghim JL (2009). A review of the antioxidant mechanisms of polyphenol compounds related to iron binding. Cell Biochem Biophys.

[34] Dai J, Mumper RJ (2010). Plant phenolics: extraction, analysis and their antioxidant and anticancer properties. Molecules.

[35] Procházková D, Boušová I, Wilhelmová N (2011). Antioxidant and prooxidant properties of flavonoids. Fitoterapia.

[36] Lemańska K, Szymusiak H, Tyrakowska B, Zieliński R, Soffers AEMF, Rietjens IMCM (2001). The influence of pH on antioxidant properties and the mechanism of antioxidant action of hydroxyflavones. Free Radic Biol Med..

[37] Firuzi O, Lacanna A, Petrucci R, Marrosu G, Saso L (2005). Evaluation of the antioxidant activity of flavonoids by “ferric reducing antioxidant power” assay and cyclic voltammetry. Biochim Biophys Acta.

[38] Ash S, De SP, Pyne S, Misra A (2010). Excited state intramolecular proton transfer in 3-hydroxy flavone and 5-hydroxy flavone: a DFT based comparative study. J Mol Model.

[39] Rice-Evans CA, Miller NJ, Paganga G (1996). Structure-antioxidant activity relationships of flavonoids and phenolic acids. Free Radic Biol Med..

[40] Kang KA, Zhang R, Chae S, Lee SJ, Kim J, Kim J (2010). Phloroglucinol (1,3,5-trihydroxybenzene) protects against ionizing radiation-induced cell damage through inhibition of oxidative stress in vitro and in vivo. Chem-Biol Interact..

[41] Chen BY, Ma CM, Liao JH, Hsu AW, Tsai PW, Wu CC (2017). Feasibility study on biostimulation of electron transfer characteristics by edible herbs-extracts. J Taiwan Inst Chem Eng..

[42] Kilmartin P, Hsu CF (2003). Characterisation of polyphenols in green, oolong, and black teas, and in coffee, using cyclic voltammetry. Food Chem.

[43] Zhang S, Qu Z, Hsueh CC, Chang CT, Chen BY (2019). Deciphering electron-shuttling characteristics of *Scutellaria baicalensis* Georgi and ingredients for bioelectricity generation in microbial fuel cells. J Taiwan Inst Chem Eng..

[44] Gao Z, Huang K, Yang X, Xu H (1999). Free radical scavenging and antioxidant activities of flavonoids extracted from the radix of *Scutellaria baicalensis* Georgi. Biochim Biophys Acta.

[45] Chen BY, Liao JH, Hsueh CC, Qu Z, Hsu AW, Chang CT (2018). Deciphering biostimulation strategy of using medicinal herbs and tea extracts for bioelectricity generation in microbial fuel cells. Energy..

[46] Danilewicz JC (2012). Review of oxidative processes in wine and value of reduction potentials in enology. Am J Enol Vitic.

[47] Makhotkina O, Kilmartin PA (2009). Uncovering the influence of antioxidants on polyphenol oxidation in wines using an electrochemical method: cyclic voltammetry. J Electroanal Chem.

[48] Zhang H, Wang T, Qiu Y, Fu FF, Yu Y (2016). Electrochemical behavior and determination of baicalin on a glassy carbon electrode modified with molybdenum disulfide nano-sheets. J Electroanal Chem.

[49] Janeiro P, Corduneanu O, Bret AMO (2005). Chrysin and (±)-taxifolin electrochemical oxidation mechanisms. Electroanalysis.

[50] Martinez CM, Alvarez LH (2018). Application of redox mediators in bioelectrochemical systems. Biotechnol Adv.

[51] Glasser NR, Saunders SH, Newman DK (2017). The colorful world of extracellular electron shuttles. Annu Rev Microbiol.

[52] Hernandez ME, Newman DK (2001). Extracellular electron transfer. Cell Mol Life Sci.

[53] Lee J (2010). Caffeic acid derivatives in dried Lamiaceae and *Echinacea purpurea* products. J Funct Foods..

[54] Bastos DHM, Saldanha LA, Catharino RR, Sawaya ACHF, Cunha IBS, Carvalho PO (2007). Phenolic Antioxidants Identified by ESI-MS from Yerba Maté (*Ilex paraguariensis*) and Green Tea (*Camelia sinensis*) Extracts. Molecules.

[55] Janicsak G, Mathe I, Miklossy-Vari V, Blunden G (1999). Comparative studies of the rosmarinic and caffeic acid contents of *Lamiaceae* species. Biochem Syst Ecol..

[56] Roby MHH, Sarhan MA, Selim KAH, Khalel KI (2013). Evaluation of antioxidant activity, total phenols and phenolic compounds in thyme (*Thymus vulgaris* L.), sage (*Salvia officinalis* L.), and marjoram (*Origanum majorana* L.) extracts. Ind Crops Prod..

[57] Yen NT, Thu NV, Zhao BT, Lee JH, Kim JA, Son JK (2014). Quantitative determination of compounds from *Akebia quinata* by high-performance liquid chromatography. Bull Korean Chem Soc.

[58] Wang M, Simon JE, Aviles IF, He K, Zheng QY, Tadmor Y (2003). Analysis of antioxidative phenolic compounds in artichoke (*Cynara scolymus* L.). J Agric Food Chem..

[59] Wong SK, Lim YY, Ling SK, Chan EWC (2014). Caffeoylquinic acids in leaves of selected Apocynaceae species: their isolation and content. Pharmacogn Res..

[60] Innocenti M, Gallori S, Giaccherini C, Ieri F, Vincieri FF, Mulinacci N (2005). Evaluation of the phenolic content in the aerial parts of different varieties of *Cichorium intybus* L. J Agric Food Chem.

[61] Wang J, Liao X, Zhang H, Du J, Chen P (2003). Accumulation of chlorogenic acid in cell suspension cultures of *Eucommia ulmoides*. Plant Cell Tissue Organ Cult.

[62] Zhang B, Yang R, Liu CZ (2008). Microwave-assisted extraction of chlorogenic acid from flower buds of *Lonicera japonica* Thunb. Sep Purif Technol.

[63] Chkhikvishvili ID, Kahrebava GI (2001). Cichoric and chlorogenic acids in plant species from Georgia. Appl Biochem Microbiol.

[64] Lee J, Scagel CF (2009). Chicoric acid found in basil (*Ocimum basilicum* L.) leaves. Food Chem..

[65] Peng R, Wu Q, Chen X, Ghosh R (2017). Purification of Danshensu from *Salvia miltiorrhiza* extract using graphene oxide-based composite adsorbent. Ind Eng Chem Res.

[66] You SP, Ma L, Zhao J, Zhang SL, Liu T (2016). Phenylethanol glycosides from *Cistanche tubulosa* suppress hepatic stellate cell activation and block the conduction of signaling pathways in tgf-β1/smad as potential anti-hepatic fibrosis agents. Molecules.

[67] Nishibe S, Okabe K, Tsukamoto H, Sakushima A, Hisada S (1982). The structure of forsythiaside isolated from *Forsythia suspense*. Chem Pharm Bull.

[68] Wishart DS, Feunang YD, Marcu A, Guo AC, Liang K, Vázquez-Fresno R, et al. HMDB 4.0—The Human Metabolome Database for 2018. http://www.hmdb.ca/metabolites/HMDB0033884. 10.1093/nar/gkx1089 PMC5753273 29140435

[69] Yannai S (2012). Dictionary of food compounds with CD-ROM: Additives, flavors, and ingredients.

[70] Anesini C, Ferraro G, López P, Borda E (2001). Different intracellular signals coupled to the antiproliferative action of aqueous crude extract from *Larrea divaricata* Cav. and nor-dihydroguaiaretic acid on a lymphoma cell line. Phytomedicine..

[71] Andary C, Wylde R, Laffite C, Privat G, Winternitz F (1982). Structures of verbascoside and orobanchoside, caffeic acid sugar esters from *Orobanche rapum*-*genistae*. Phytochemistry.

[72] Lu J, Pu X, Li Y, Zhao Y, Tu G (2005). Bioactive phenylpropanoid glycosides from *Buddleia lindleyana*. Z Nat forsch..

[73] Guedon DJ, Pasquier BP (1994). Analysis and distribution of flavonoid glycosides and rosmarinic acid in 40 *Mentha* × *piperita* clones. J Agric Food Chem.

[74] Erkan N, Ayranci G, Ayranci E (2008). Antioxidant activities of rosemary (*Rosmarinus officinalis* L.) extract, blackseed (*Nigella sativa* L.) essential oil, carnosic acid, rosmarinic acid and sesamol. Food Chem..

[75] Ai CB, Li LN (1988). Stereostructure of salvianolic acid B and isolation of salvianolic acid C from *Salvia miltiorrhiza*. J Nat Prod.

[76] Li L, Tan R, Chen W (1984). Salvianolic Acid A, a new depside from roots of *Salvia miltiorrhiza*. Planta Med.

[77] Dong J, Liu Y, Liang Z, Wang W (2010). Investigation on ultrasound-assisted extraction of salvianolic acid B from *Salvia miltiorrhiza* root. Ultrason Sonochem..

[78] Kobayashi H, Oguchi H, Takizawa N, Miyase T, Ueno A, Usmanghani K (1987). New phenylethanoid glycosides from *Cistanche tubulosa* (SCHRENK) HOOK. f. I. Chem Pharm Bull..

[79] Pardo F, Perich F, Villarroel L, Torres R (1993). Isolation of verbascoside, an antimicrobial constituent of *Buddleja globosa* leaves. J Ethnopharmacol.

[80] Avila GJ, De Liverant JG, MartíNez A, MartíNez G, Muñoz JL (1999). Mode of action of *Buddleja cordata* verbascoside against *Staphylococcus aureus*. J Ethnopharmacol.

[81] Schlauer J, Budzianowski J, Kukułczanka K, Ratajczak L (2004). Acteoside and related phenylethanoid glycosides in *Byblis liniflora Salisb.* Plants propagated in vitro and its systematic significance. Acta Soc Bot Pol..

[82] Oyourou JN, Combrinck S, Regnier T, Marston A (2013). Purification, stability and antifungal activity of verbascoside from *Lippia javanica* and *Lantana camara* leaf extracts. Ind Crop Prod..

[83] Saimaru H, Orihara Y (2010). Biosynthesis of acteoside in cultured cells of *Olea europaea*. J Nat Med.

[84] Sarkhail P, Nikan M, Sarkheil P, Gohari AR, Ajani Y, Hosseini R (2014). Quantification of verbascoside in medicinal species of *Phlomis* and their genetic relationships. DARU J Pharm Sci..

[85] Murai M, Tamayama Y, Nishibe S (1995). Phenylethanoids in the herb of *Plantago lanceolate* and inhibitory effect on arachidonic acid-induced mouse ear edema. Planta Med.

[86] Speranza L, Franceschelli S, Pesce M, Menghini L, Patruno A, Vinciguerra I (2009). Anti-inflammatory properties of the plant *Verbascum mallophorum*. J Biol Regul Homeost Agents.

[87] Georgiev M, Ali K, Alipieva K, Verpoorte R, Choi YH (2011). Metabolic differentiations and classification of *Verbascum* species by NMR-based metabolomics. Phytochemistry.

[88] Deepak M, Handa SS (2000). Antiinflammatory activity and chemical composition of extracts of *Verbena officinalis*. Phytother Res..

[89] Fukui H, Hasan AFMF, Ueoka T, Kyo M (1998). Formation and secretion of a new brown benzoquinone by hairy root cultures of *Lithospermum erythrorbizon*. Phytochemistry.

[90] Pavunraj M, Muthu C, Ignacimuthu S, Janarthanan S, Duraipandiyan V, Raja N (2011). Antifeedant activity of a novel 6-(4,7-hydroxy-heptyl) quinone^®^ from the leaves of the milkweed *Pergularia daemia* on the cotton bollworm *Helicoverpa armigera* (Hub.) and the tobacco armyworm *Spodoptera litura* (Fab.). Phytoparasitica.

[91] Morimoto M, Fujii Y, Komai K (1999). Antifeedants in Cyperaceae: coumaran and quinones from *Cyperus* spp. Phytochemistry.

[92] Kupchan SM, Obasi ME (1960). A note on the occurrence of 2,6-dimethoxy- benzoquinone in *Rauwolfia vomitoria*. J Am Pharm Assoc..

[93] Jones E, Ekundayo O, Kingston DGI (1981). Plant anticancer agents. XI. 2,6-dimethoxybenzoquinone as a cytotoxic constituent of *Tibouchina pulchra*. J Nat Prod.

[94] Radhakrishnan N, Gnanamani A, Mandal AB (2011). A potential antibacterial agent Embelin, a natural benzo-quinone extracted from *Embelia ribes*. Biol Med..

[95] Link KP, Walker JC (1933). The isolation of catechol from pigmented onion scales and its significance in relation to disease resistance in onions. J Biol Chem.

[96] Link KP, Angell HR, Walker JC (1929). The isolation of protocatechuic acid from pigmented onion scales and its significance in relation to disease resistance in onions. J Biol Chem.

[97] Mukherjee KS, Ghosh PK, Badruddoza S (1981). Diterpenoid quinones of *Salvia lanata*. Phytochemistry.

[98] Borges-Argáez R, Uc-Cachón A, Quintal-Novelo C, Canché-Escamilla G, Farfán MC (2013). A selective chemical method for the separation of quinones from the stem bark of *Diospyros anisandra*. Int J Curr Pharm Res..

[99] Lajubutu BA, Pinney RJ, Roberts MF, Odelola HA, Oso BA (1995). Antibacterial activity of diosquinone and plumbagin from the root of *Diospyros mespiliformis* (Hostch) (Ebenaceae). Phytother Res..

[100] Fujita Y, Hara Y, Suga C, Morimoto T (1981). Production of shikonin derivatives by cell suspension cultures of *Lithospermum erythrorhizon* II. A new medium for the production of shikonin derivatives. Plant Cell Rep..

[101] The Office of Dietary Supplements (ODS). National Institutes of Health. https://ods.od.nih.gov/factsheets/vitaminK-HealthProfessional/.

[102] Yadav JP, Arya V, Yadav S, Panghal M, Kumar S, Dhankhar S (2010). *Cassia occidentalis* L.: a review on its ethnobotany, phytochemical and pharmacological profile. Fitoterapia..

[103] Dave H, Ledwani L. A review on anthraquinones isolated from *Cassia* species and their applications. Indian J Nat Prod Res. 2012;291–319.

[104] Kaur P, Chandel M, Kumar S, Kumar N, Singh B, Kaur S (2010). Modulatory role of alizarin from *Rubia cordifolia* L. against genotoxicity of mutagens. Food Chem Toxicol..

[105] Lodhi AH, Sant’Ana AEG, Charlwood BV (1994). Quantitative analysis of alizarin in tissue cultures of *Rubia* species by high performance liquid chromatography. Phytochem Anal.

[106] Gautam R, Karkhile KV, Bhutani KK, Jachak SM (2010). Anti-inflammatory, cyclooxygenase (COX)-2, COX-1 Inhibitory, and free radical scavenging effects of *Rumex nepalensis*. Planta Med.

[107] Ng TB, Liu F, Lu Y, Cheng CH, Wang Z (2003). Antioxidant activity of compounds from the medicinal herb *Aster tataricus*. Comp Biochem Physiol C Toxicol Pharmacol.

[108] Yang YC, Lim MY, Lee HS (2003). Emodin isolated from *Cassia obtusifolia* (Leguminosae) seed shows larvicidal activity against three mosquito species. J Agric Food Chem.

[109] Kremer D, Kosalec I, Locatelli M, Epifano F, Genovese S, Carlucci G (2012). Anthraquinone profiles, antioxidant and antimicrobial properties of *Frangula rupestris* (scop.) schur and *Frangula alnus* mill. bark. Food Chem..

[110] Chao PM, Kuo YH, Lin YS, Chen CH, Chen SW, Kuo YH (2010). The metabolic benefits of *Polygonum hypoleucum* Ohwi in HepG2 Cells and Wistar rats under lipogenic stress. J Agric Food Chem.

[111] Ghosh S, Das Sarma M, Patra A, Hazra B (2010). Anti-Inflammatory and anticancer compounds isolated from *Ventilago madraspatana* Gaertn., *Rubia cordifolia* Linn. and *Lantana camara* Linn. J Pharm Pharmacol..

[112] Nian SH, Li HJ, Liu EH, Li P (2017). Comparison of α-glucosidase inhibitory effect and bioactive constituents of *Anemarrhenae rhizoma* and fibrous roots. J Pharm Biomed Anal.

[113] Mischenko NP, Fedoreyev SA, Glazunov VP, Tchernoded GK, Bulgakov VP, Zhuravlev YN (1999). Anthraquinone production by callus cultures of *Rubia cordifolia*. Fitoterapia.

[114] Dinda B, Das SK, Hajra AK, Bhattacharya A, De K, Chel G, et al. Chemical constituents of *Plumbago indica* roots and reactions of plumbagin: Part II. Indian J Chem. 1999;577–82.

[115] Willför S, Mumtaz A, Karonen M, Reunanen M, Mohammad A, Harlamow R (2009). Extractives in bark of different conifer species growing in Pakistan. Holzforschung.

[116] Shen D, Wu Q, Wang M, Yang Y, Lavoie EJ, Simon JE (2006). Determination of the predominant catechins in *Acacia catechu* by liquid chromatography/electrospray ionization-mass spectrometry. J Agric Food Chem.

[117] Yilmaz Y, Toledo RT (2004). Major flavonoids in grape seeds and skins: antioxidant capacity of catechin, epicatechin, and gallic acid. J Agric Food Chem.

[118] Dalluge JJ, Nelson BC (2000). Determination of tea catechins. J Chromatogr A.

[119] Aizpurua-Olaizola O, Ormazabal M, Vallejo A, Olivares M, Navarro P, Etxebarria N (2015). Optimization of supercritical fluid consecutive extractions of fatty acids and polyphenols from *Vitis vinifera* grape wastes. J Food Sci.

[120] Zhang A, Chan PT, Luk YS, Ho WKK, Chen ZY (1997). Inhibitory effect of jasmine green tea epicatechin isomers on LDL-oxidation. J Nutr Biochem.

[121] Zuo Y, Chen H, Deng Y (2002). Simultaneous determination of catechins, caffeine and gallic acids in green, Oolong, black and pu-erh teas using HPLC with a photodiode array detector. Talanta.

[122] Vetrova EV, Maksimenko EV, Borisenko SN, Lekar AV, Borisenko NI, Minkin VI (2017). Extraction of rutin and quercetin antioxidants from the buds of *Sophora japonica* L. by subcritical water. Russ J Phys Chem B..

[123] Zou QY, Wu HF, Tang YL, Chen DZ (2016). A new labdane diterpene from the rhizomes of *Alpinia officinarum*. Nat Prod Res.

[124] Leung LK, Su Y, Chen R, Zhang Z, Huang Y, Chen ZY (2001). Theaflavins in black tea and catechins in green tea are equally effective antioxidants. J Nutr.

[125] Fang J (2015). Classification of fruits based on anthocyanin types and relevance to their health effects. Nutrition..

[126] Daniel EM, Krupnick AS, Heur YH, Blinzler JA, Nims RW, Stoner GD (1989). Extraction, stability, and quantitation of ellagic acid in various fruits and nuts. J Food Compost Anal..

[127] Chen BY, Liao JH, Hsu AW, Tsai PW, Hsueh CC (2018). Exploring optimal supplement strategy of medicinal herbs and tea extracts for bioelectricity generation in microbial fuel cells. Bioresour Technol.

[128] Li J, Romero-Garcia R, Suckling J, Feng L (2019). Habitual tea drinking modulates brain efficiency: evidence from brain connectivity evaluation. Aging..

[129] Janeiro P, Brett AMO (2004). Catechin electrochemical oxidation mechanisms. Anal Chim Acta.

[130] Xu B, Lan JC, Sun Q, Hsueh CC, Chen BY (2019). Deciphering optimal biostimulation strategy of supplementing anthocyanin-abundant plant extracts for bioelectricity extraction in microbial fuel cells. Biotechnol Biofuels.

[131] Moran JJ, Whitmore LM, Isern NG, Romine MF, Riha KM, Inskeep WP (2016). Formaldehyde as a carbon and electron shuttle between autotroph and heterotroph populations in acidic hydrothermal vents of Norris Geyser Basin, Yellowstone National Park. Extremophiles.

[132] Shang X, Pan H, Li M, Miao X, Ding H (2011). *Lonicera japonica* Thunb.: ethnopharmacology, phytochemistry and pharmacology of an important traditional Chinese medicine. J Ethnopharmacol..

[133] Gil ES, Couto RO (2013). Flavonoid electrochemistry: a review on the electroanalytical applications. Rev Bras Farmacogn.

[134] Ferreira D, Marais JPJ, Coleman CM, Slade D (2003). Phytochemistry of the mopane, *Colophospermum mopane*. Phytochemistry.

[135] Ferreira D, Marais JPJ, Coleman CM, Slade D. Proanthocyanidins: chemistry and biology. In: Liu HW, Mander L, editors. Comprehensive natural products II: chemistry and biology, vol. 6. Elsevier Science; 2010. p. 605–61.

[136] Castañeda-Ovando A, de Lourdes Pacheco-Hernández M, Páez-Hernández ME, Rodríguez JA, Galán-Vidal CA (2009). Chemical studies of anthocyanins: a review. Food Chem..

[137] Do QD, Angkawijaya AE, Nguyen PLT, Huynh LH, Ju YH (2014). Effect of extraction solvent on total phenol content, total flavonoid content, and antioxidant activity of *Limnophila aromatica*. J Food Drug Anal..

[138] Walczak MM, Dryer DA, Jacobson DD, Foss MG, Flynn NT (1997). pH-Dependent redox couple: illustrating the nernst equation using cyclic voltammetry. J Chem Educ.

[139] Wedege K, Dražević E, Konya D, Bentien A (2016). Organic redox species in aqueous flow batteries: redox potentials, chemical stability and solubility. Sci Rep..

[140] Hoover K, Kishida KT, Digiorgio LA, Workman J, Alaniz SA, Hammock BD (1998). Inhibition of baculoviral disease by plant-mediated peroxidase activity and free radical generation. J Chem Ecol.

[141] Karaosmanoglu H, Suthanthangjai W, Travas-Sejdic J, Kilmartin PA (2016). Electrochemical analysis of beverage phenolics using an electrode modified with poly(3,4-ethylenedioxithiophene). Electrochim Acta..

[142] Abdel-Hamid R, Newair EF, Garcia F (2016). Electroanalytical determination of luteolin in peanut hulls using carbon black modified glassy carbon electrode. Br J Anal Chem..

[143] Vellaichamy B, Ponniah SK, Periakaruppan P (2017). An in situ synthesis of novel Au@NG-PPy nanocomposite for enhanced electrocatalytic activity toward selective and sensitive sensing of catechol in natural samples. Sens Actuator B-Chem.

[144] Feng C, Ma L, Li F, Mai H, Lang X, Fan S (2010). A polypyrrole/anthraquinone-2,6-disulphonic disodium salt (PPy/AQDS)-modified anode to improve performance of microbial fuel cells. Biosens Bioelectron.

[145] Tang X, Li H, Du Z, Ng HY (2014). Spontaneous modification of graphite anode by anthraquinone-2-sulfonic acid for microbial fuel cells. Bioresour Technol.

[146] Pei J, Chen A, Zhao L, Cao F, Ding G, Xiao W (2017). One-pot synthesis of hyperoside by a three-enzyme cascade using a UDP-galactose regeneration system. J Agric Food Chem.

